# Rapid Downregulation of DAB2 by Toll-Like Receptor Activation Contributes to a Pro-Inflammatory Switch in Activated Dendritic Cells

**DOI:** 10.3389/fimmu.2019.00304

**Published:** 2019-02-27

**Authors:** Vanessa Figliuolo da Paz, Deepa R. Jamwal, Michael Gurney, Monica Midura-Kiela, Christy A. Harrison, Christopher Cox, Jean M. Wilson, Fayez K. Ghishan, Pawel R. Kiela

**Affiliations:** ^1^Department of Pediatrics, University of Arizona, Tucson, AZ, United States; ^2^Department of Cellular and Molecular Medicine, University of Arizona, Tucson, AZ, United States; ^3^Department of Immunobiology, University of Arizona, Tucson, AZ, United States

**Keywords:** Dab2, dendritic cells, immunoregulation, small intestine, colon, inflammation

## Abstract

Dendritic cells (DCs) are pivotal in regulating tolerogenic as well as immunogenic responses against microorganisms by directing both the innate and adaptive immune response. In health, phenotypically different DC subsets found in the gut mucosa are maintained in their tolerogenic state but switch to a pro-inflammatory phenotype during infection or chronic autoinflammatory conditions such as inflammatory bowel disease (IBD). The mechanisms that promote the switch among the mucosal DCs from a tolerogenic to an immunogenic, pro-inflammatory phenotype are incompletely understood. We hypothesized that disabled homolog 2 (DAB2), recently described as a negative regulator of DC immunogenicity during their development, is regulated during intestinal inflammation and modulates mucosal DC function. We show that DAB2 is highly expressed in colonic CD11b^+^CD103^−^ DCs, a subset known for its capacity to induce inflammatory Th1/Th17 responses in the colon, and is downregulated predominantly in this DC subset during adoptive T cell transfer colitis. Administration of Dab2-deficient DCs (DC2.4^*Dab2*−*/*−^ cells) modulated the course of DSS colitis in wild-type mice, enhanced mucosal expression of *Tnfa, Il6*, and *Il17a*, and promoted neutrophil recruitment. In bone-marrow derived dendritic cells (BMDC), DAB2 expression correlated with CD11b levels and DAB2 was rapidly and profoundly inhibited by TLR ligands in a TRIF- and MyD88-dependent manner. The negative modulation of DAB2 was biphasic, initiated with a quick drop in DAB2 protein, followed by a sustained reduction in *Dab2* mRNA. DAB2 downregulation promoted a more functional and activated DC phenotype, reduced phagocytosis, and increased CD40 expression after TLR activation. Furthermore, *Dab2* knockout in DCs inhibited autophagy and promoted apoptotic cell death. Collectively, our results highlight the immunoregulatory role for DAB2 in the intestinal dendritic cells and suggest that DAB2 downregulation after microbial exposure promotes their switch to an inflammatory phenotype.

## Introduction

Dendritic cells (DCs) are pivotal in the immune response against foreign invaders. They interact with other innate immune cells and prime adaptive immunity to determine the type, specificity, and duration of the T cell mediated immune responses (1). DCs can recognize pathogen-associated molecular patterns (PAMPs) in large part via Toll-like receptors (TLR), which bind to a large range of molecules produced by bacteria, viruses, and fungi. TLRs (1–9 and 11–13) are expressed on the plasma and organellar membranes of murine DCs (2), and their PAMP binding activates NF-kB/AP-1- or IRF-3-dependent pathways, which ultimately drive DC maturation and cytokine production/release (3). As a consequence, DCs phagocytose the invaders, and mature while migrating to the draining lymph nodes where they present the processed antigen to T lymphocytes. DC maturation involves reduced endocytic activity, up-regulation of co-stimulatory molecules, adhesion molecules, chemokine receptors, and secretion of inflammatory cytokines (4). The signal provided by activated DCs leads to T cell expansion and the polarization toward the T helper phenotypes following exposure to different cytokine combinations. Th1 cells develop in the presence of IL-12, IL-18, and IL-23, while Th2 requires IL-4 or IL-5 and Th17 (IL-6, Il-1β, IL-23, and TGFβ) (5, 6). In addition to potentiating DC function, TLR signaling is involved in cell death and survival. An increase in anti-apoptotic proteins is observed after DC activation (7), which promotes T cell priming, but after antigen presentation, DCs are eliminated through apoptosis as a mechanism to regulate the number of activated T cells and immune response resolution (8).

A healthy mucosal immune system has the important role of promoting immune tolerance to avoid specific immune responses against the large load of commensal bacterial and dietary antigens. Likely through a combination of epithelial injury and genetic predisposition, a disturbed tolerance in the intestinal mucosa leads or contributes to chronic Crohn's disease (CD) or ulcerative colitis (UC) when the secretion of an array of inflammatory mediators leads to tissue damage, pain, and compromised function (9). The immune response against the luminal microbiota is the main cause of an exacerbated intestinal immune response in patients with inflammatory bowel diseases (IBD). Transfer of syngeneic naïve T cells into immunodeficient (Rag2^−/−^) mice is sufficient to induce IBD-like intestinal inflammation (10). The mucosa of CD patients, as well as in mice with adoptive T cell transfer colitis, is infiltrated by a mix of IL-17A^+^ and IFN-γ^+^ T cells (11). Due to their ability to recognize PAMPs and to ultimately drive T cell priming and differentiation, DCs are implicated in IBD pathogenesis. Activated DCs accumulate at the sites of inflammation in human IBD and in murine models of colitis (12, 13). Although DCs promote mucosal homeostasis in the steady state, DCs from the tissues of IBD patients are phenotypically and functionally distinct from those involved in immune tolerance (14). They express increased levels of activation markers (MHCII and co-stimulatory molecules), chemokine receptors, and have enhanced TLR responsiveness (15, 16). In the murine gut, CD103^+^CD11b^−^ DCs, although also capable of instructing IFNγ^+^ T cells (17), have been credited with a major role in the maintenance of mucosal tolerance, in part due to their ability to produce retinoic acid necessary for the development of FoxP3^+^ iTregs (18, 19). CD11b^+^ DC subsets contribute to inflammation via selective expansion of Th17 cells (CD103^+^CD11b^+^) or both IFNγ^+^ and IL-17^+^ T cells (CD103^−^CD11b^+^ DCs) (17). Although a lot has been clarified about the function of the different intestinal DCs during intestinal inflammation, the mechanisms that promote the switch among the mucosal DCs from a tolerogenic to an immunogenic, pro-inflammatory phenotype are not fully understood.

Disabled homolog 2 (Dab2), is a clathrin and cargo binding endocytic adaptor protein which modulates multiple cellular signaling pathways, including Ras/MAPK and TGFβ (20–22). Dab2 is expressed in at least two isoforms, p96 or p67. The ninth coding exon corresponding to the amino acids 230–447 of p96-Dab2 enables the interaction with membrane receptors and its identification as an endocytic protein (23). First cloned from CSF-1-treated mouse macrophages (24), Dab2 has been identified as an adaptor protein for the low-density lipoprotein receptor (LDLR) (25), type II transforming growth factor b receptor (TGFbR) (22) and integrin β1 (26), among others, and has become recognized as a key player in the receptor trafficking in different systems. Null knockout in mice is embryonically lethal with a defect in the visceral endoderm development (27). In immune cells, Dab2 was identified as a target gene of FOXP3, critical for the *in vitro* and *in vivo* function of Tregs; Tregs lacking Dab2 were dysfunctional and unable to efficiently control colitogenic T cells in an adoptive transfer model (28). Among the innate immune cells, Dab2 is highly expressed in macrophages, where it plays an important role in macrophage polarization, activation, and inflammation. Dab2 repression in macrophages contributes to a pro-inflammatory profile after exposure to TLR stimulation, and exacerbates adipose tissue inflammation induced by chronic high-fat feeding (29). Dab2 expression is believed to contribute to an immune tolerant phenotype in macrophages by acting as a negative immune regulator of TRAF-6 and NF-kB activation (29), and by inhibiting TRIF-mediated cell signaling triggered after TLR4 activation and endocytosis (30). The anti-inflammatory phenotype in peritoneal macrophages correlated with increased Dab2 expression (31). More recently, Dab2 downregulation in macrophages was implicated in more pronounced liver damage in Ldlr^−/−^ mice fed a Western diet, a murine model of arteriosclerosis (32). In DCs, Dab2 was described as a negative regulator of their immunogenicity during DC development (33), but the control of its expression in intestinal dendritic and its contribution to intestinal immune tolerance or immunity has not been explored.

Here, we describe that Dab2 is highly expressed in colonic CD11b^+^CD103^−^ DCs and downregulated in the same cell type during experimental colitis. The high expression of Dab2 in CD11b^+^CD103^−^ cells may be a critical suppressive mechanism to limit the immune responses against the high load of commensal microbial antigens in this segment of the gut. In support of this hypothesis, we show that Dab2 downregulation in DCs was triggered by TLR agonists in a biphasic fashion: through initial rapid reduction of Dab2 protein independent of lysosomal and proteasome degradation, followed by a significant decrease in Dab2 mRNA. We further show that Dab2 downregulation impacts a key step of DC function and activation, such as phagocytosis, CD40 expression and cytokine production, and promotes cell death while reducing autophagy. Our results contribute to the understanding of DC participation in the intestinal homeostasis and inflammation, describe a new player in the DC physiology and immune response and suggest that Dab2 downregulation after microbial exposure favors an inflammatory phenotype in intestinal DCs.

## Materials and Methods

### Mice

Male C57BL/6J-*Ticam1*^*Lps*2^/J (TRIF^−/−^), B6.129P2(SJL)-*Myd88*^*tm*1.1*Defr*^/J (MyD88^−/−^), B6(Cg)-*Rag2*^*tm*1.1*Cgn*^/J (Rag2^−/−^) and C57BL/6J mice, 6–8 weeks old, were obtained from Jackson Laboratory. The mice were housed under controlled conditions in the Specific Pathogen Free animal facility in the Bio-5 Institute at the University of Arizona. All procedures performed at the University of Arizona were handled in accordance with University of Arizona Animal Care (UAC) guidelines and approved by Animal Care and Use Committee (protocol # 07-126).

### Lamina Propria Isolation

To isolate intestinal leukocytes, small intestine or colon were flushed with Ca^2+^/Mg^2+^-free (CMF) Hank's balanced salt solution (HBSS) (Gibco) and the Peyer Patch was excised. The intestines were opened longitudinally, washed in HBSS and cut into 1 cm segments. The intestinal fragments were then incubated in HBSS 10 mM HEPES 2% FBS (HHF) containing 2 mM EDTA at 37°C with shaking for 20 min and vigorously vortexed for 1 min. Supernatants were discarded and intestinal fragments were digested for 20 min with 100 U/mL type 1 collagenase (Worthington Biochemical Corporation) in HHF containing 40 μg/mL DNase I (Gold Biotechnology USA) at 37°C while shaking. The fragments were vortexed vigorously for 1 min and the cell suspensions passed through 100 μm filter (BD Falcon) and collected in complete RPMI 1640 medium containing 2 mM l-glutamine, 100 μg/mL penicillin, 100 μg/mL streptomycin, 10% FBS (all Gibco, Invitrogen). Viable cells were counted using trypan blue exclusion and hemocytometer and adjusted in 2%FBS, 0.01% sodium azide PBS (FACS buffer) to stain for flow cytometry.

### Generation and Treatment of Bone-Marrow Derived Dendritic Cells (BMDCs)

Bone marrow (BM) was obtained from mouse femurs and tibias after flushing with cold complete RPMI medium. Red blood cells were lysed using RBC lysis buffer (BD Biosciences) and the leukocytes were incubated with 200 ng/mL FLT3L (Biolegend) in complete RPMI 1640 medium containing 55 μM β-mercaptoethanol (Gibco) for 7 days at 37°C 5% CO_2_, followed by additional 2 days incubation with complete RPMI 1640 containing 20 ng/mL GM-CSF (Peprotech) and 200 ng/mL FLT3L (Biolegend) to generate CD103^+^DC. BMDCs were harvested for further analysis on day 9, when their phenotype was determined by flow cytometry. To investigate the impact of TLR signaling on Dab2 expression, BMDCs from wild type C57BL/6 mice, TRIF^−/−^ or MyD88^−/−^ mice were incubated overnight with TLR1-9 agonists (InvivoGen). BMDCs were also treated for a short period with 100 ng/mL LPS (InvivoGen) or pre-treated with 100 nM bafilomycin A1 (Sigma Aldrich) or 2 μM MG132 (Sigma Aldrich) for 30 min before treatment with 100 ng/mL LPS for 1 h. To study Dab2 protein and mRNA stability, BMDCs were treated with 5 μg/mL cycloheximide (Sigma Aldrich) or 10 μg/mL actinomycin D (Santa Cruz Biotechnology) for different durations. Samples were prepared for western blotting or for qRT-PCR. RNA isolation from BMDCs was performed using Isolate II Mini kit (Bioline) and cDNA synthesis using SensiFAST™ cDNA synthesis kit (Bioline) according to the manufacturer's instructions. The primers used are listed on Table S1.

### Human Dendritic Cell Differentiation

Human dendritic cells were differentiated from human blood monocytes using the Human Monocyte-derived Dendritic Cell Differentiation kit (R&D Systems). Briefly, peripheral blood mononuclear cells were isolated from human blood (IRB approval # Project, No. *1711026297*) followed by isolation of monocytes using Microbeads for human CD14^+^ isolation (Miltenyi Biotec), and incubation of 1 × 10^6^ CD14^+^ cells/mL in differentiation media for 7 days at 37°C 5%CO_2_. Immature dendritic cells were incubated with 100 ng/mL LPS for 48 h and DAB2 expression assessed by Western blotting.

### Induction of T-Cell Dependent Colitis

CD4^+^ T cells were enriched from the spleens of male C57BL/6J mice using the EasySep™ isolation kit (STEMCELL Technologies) and resuspended in 0.5%FBS 2 mM EDTA in phosphate-buffered saline (PBS) (MACS buffer). CD45RB^high^CD4^+^T cells were subsequently flow-sorted at the University of Arizona Cytometry Core Facility using BD FACSAriaIII and FACSDiva software (BD Biosciences). The resulting naïve T cells were resuspended in sterile PBS and 5 × 10^5^ cells were intraperitoneally (i.p.) injected into Rag2^−/−^ male C57BL/6J mice. Body weight was monitored weekly and the recipient and control groups (injected with PBS only) were sacrificed at the time of an approximate 20% weight loss (9 weeks post transfer). Colitis severity was evaluated by cytokine expression using qRT-PCR (the primers used are listed in Table S1) and expressed relative to the gene of reference, the TATA-box binding protein (TBP).

### Establishment of Stable Dendritic Cell Knockout for Dab2 Expression

Immortalized mouse dendritic cell line DC2.4 (34) was used to establish a model of *Dab2* deficiency using *Dab2* Double Nickase Plasmid (Santa Cruz Biotechnology), with subsequent selection using antibiotics and clonal selection. Briefly, DC2.4 cells were plated at a density of 5 × 10^5^ cells/well on a 6-well plate for 24 h in complete DMEM containing 2 mM l-glutamine, 100 μg/mL penicillin, 100 μg/mL streptomycin, 10% FBS (all Gibco, Invitrogen), and transfected with 2.0 μg of Dab2 Double Nickase Plasmid in transfection media. After 48 h, the GFP^+^ cells were sorted using FACSAriaIII cytometer and FACSDiva software (BD Biosciences), and the cells were kept in 6-well plates containing complete DMEM until ca. 80% confluence when they were moved to complete DMEM containing 7.5 μg/mL Puromycin (Sigma Aldrich). The cells were kept under selection for 8 days, and the media was replaced with freshly prepared selective media every 3 days. Cell cloning was performed by serial dilution in a 96-well plate containing selective media and stable *Dab2* knockout cells lines were identified after screening by western-blot to detect DAB2 protein. DC2.4^WT^ or DC2.4^*Dab2*−*/*−^ cells (clones 1 and 2) used in our experiments were kept in complete DMEM for up to 20 passages.

### DSS Colitis and Transfer of WT and Dab2 Knockout DC2.4 Cells

Colitis in mice was induced in wild-type C57BL/6 mice with 3.0% (w/v) dextran sodium sulfate (DSS; 40–50 kDa, USB Corporation, Cleveland, OH, USA) provided *ad libitum* in drinking water for 8 days, at which time, mice were switched to regular water. Mice were injected i.p. with 8.0 × 10^5^ DC2.4^WT^ or DC2.4^*Dab2*−*/*−^ cells suspended in PBS 2 days after beginning DSS treatment. Body weight was monitored every 2 days and the recipient and control groups (injected with PBS only) were sacrificed on day 4 or 9. Fragments of proximal and distal colon were removed, RNA isolated, and analyzed by qRT-PCR, as described above. Additionally, distal colon fragments were collected, fixed in buffered 10% formaldehyde and embedded in paraffin. Slices of 5 μm were processed for histology using Hematoxilin and Eosin staining to visualize the intestinal architecture and inflammation.

### Flow Cytometry

Following incubation with purified anti-CD16/CD32 (eBioscience) for 10 min at 4°C, 1–5 × 10^6^ cells were stained at 4°C in the dark as recommended by the manufacturer and analyzed using an LSRII Fortessa (BD Biosciences) and FlowJo software (Tree Star). Fixable Viability Dye eFluor™ 506 (1:1,000) was used to label dead cells along with the incubation with antibodies. For intracellular staining of Dab2, cells were incubated in Fixation/Permeabilization Buffer for 30 min., treated with rabbit anti-Dab2 (1:100) (Cell Signaling) diluted in permeabilization buffer for 1 h at room temperature, then treated with Alexa Fluor 647-conjugated secondary antibody (F(ab')2-Goat anti-Rabbit IgG (H+L) (1:1,000; InvivoGen) diluted in permeabilization buffer for 30 min. The antibodies used for flow cytometry are listed in Table S2.

### Cell Treatment and Western Blotting Analysis

BMDCs were treated overnight with TLR1-9 agonists (InvivoGen) or treated with 100 ng/mL LPS for different durations, and washed in PBS before lysis for SDS-PAGE and Western blotting. For some experiments cells were pre-incubated with 100 nM bafilomycin (30 min), 50 μM MG132 (30 min) or 2 mM NAC (15 min) (all from Sigma Aldrich) before adding LPS or 55 μM THBP for 1 h. BMDCs or DC2.4 cells were disrupted and homogenized in radioimmunoprecipitation assay buffer (RIPA buffer) containing 50 mM Tris-HCl (pH 8.0), 150 mM NaCl, 1% NP-40, 0.1% SDS, 0.5% Na-deoxycholate and Halt protease and phosphatase inhibitor cocktail (ThermoFisher Scientific). Protein concentration was determined using a BCA Protein Assay (ThermoFisher Scientific) and samples were denatured and reduced in SDS-PAGE sample buffer (supplemented with 10% β-Mercaptoethanol) by heating to 95°C for 5 min. Samples (20 μg/well) were separated on a 10% Tris-Glycine protein gel (BioRad) for higher molecular weight protein detection (>30 kDa) and transferred onto nitrocellulose membranes. When analyzing lower molecular weight protein (< 30 kDa) a 4–20% gradient Tris-Glycine protein gel (BioRad) was used and samples were subsequently transferred to a polyvinylidene difluoride (PVDF) membrane. The blots were probed with primary antibodies (Table S3) (diluted 1:1,000 in 5% nonfat milk or BSA) overnight at 4°C, then with an HRP-conjugated anti-rabbit/mouse IgG secondary antibody (1:5,000) (Cell Signaling). Blots were developed using ECL Western Blotting Substrate (Thermo Scientific) and chemiluminescence was detected using the Syngene G:BOX Imaging System and GeneSys software. Densitometric analysis was performed using ImageJ software and the protein expression was normalized using β-actin (BMDCs) or GAPDH (DC2.4 cells) as an internal control.

### Immunofluorescence

Sterile coverslips (Microscope Cover Glass; Fisher) pre-treated with 1M HCl were placed in a 24-well plate and DC2.4^WT^ or DC2.4^*Dab2*−*/*−^ cells were seeded and grown in complete DMEM for at least 24 h before the assay. Cells were then treated with 100 ng/mL LPS or PBS for 20 min. Cells were washed with PBS, fixed using 4% paraformaldehyde for 15 min at room temperature (RT) and quenched with 50 mM NH_4_Cl for 10 min. Cells were permeabilized using 0.1% Triton in PBS for 10 min and incubated with blocking buffer (5% goat serum, 0.3% Triton in PBS) for 1 h at room temperature. The incubation with primary antibodies diluted in staining buffer (1% BSA 0.3% Triton in PBS) was performed overnight at 4°C in the following dilutions: mouse anti-EEA1 1:50 (sc-137130, Santa Cruz Biotechnology), rat anti-LAMP1 1:500 (ab25245, Abcam Biotechnology), or rabbit anti-Dab2 1:1,000 (12906, Cell Signaling Technology). The incubation with the following secondary antibodies was performed in staining buffer for 60 min at RT: goat anti-mouse Alexa Fluor 568 (A11019), goat anti-rat Alexa Fluor 568 (A11077), goat anti-rabbit Alexa Fluor 488 (A11070) (all from ThermoFisher Scientific). Pro-Long Gold (Life Technologies) was added to the coverslips and images were acquired with an Olympus FluoView 1200 confocal microscope (Olympus, Tokyo, Japan) with a 40 × objective. Images were assembled in Adobe Photoshop (Adobe, San Jose, CA).

### Inflammatory Phenotype in DC2.4 Cells

DC2.4^WT^ or DC2.4^*Dab2*−*/*−^ cells were incubated with 100 ng/mL LPS in complete DMEM for 24 h and analyzed by flow cytometry using antibodies against the selected surface markers listed in the Table S2. To evaluate the effects of Dab2 knockout on cytokine expression in DC2.4 cells, DC2.4^WT^, or DC2.4^*Dab2*−*/*−^ cells were incubated with 100 ng/mL LPS in complete DMEM for 6 h. The cells were washed with PBS and processed for qRT-PCR, as described above.

### Phagocytosis

DC2.4^WT^ or DC2.4^*Dab2*−*/*−^ cells (10^5^) were incubated for 1 h with or without 10 μM cytochalasin D (Sigma Aldrich) in complete media. pHrodo Red E. coli Bioparticles Conjugate for Phagocytosis were added to cells according to the manufacturer instructions (Invitrogen, Carlsbad, CA) and incubated for an additional 1 h at 37°C. Phagocytosis was also assessed in DC2.4^WT^ or DC2.4^*Dab2*−*/*−^ cells after incubation of 10^5^ cells with IgG-coated FluoSpheres carboxylate (2.0 μm, red 580/605) (Life Technologies) at 1:25 ratio for 1 h in complete DMEM. Cells were washed with PBS prior to acquisition using an LSRII Fortessa (BD Biosciences). The data was analyzed for the median fluorescence intensity (MFI) and the percentage of phagocytic cells using FlowJo (Tree Star).

### Cell Death and Autophagy

DC2.4^WT^ or DC2.4^*Dab2*−*/*−^ cells were seeded in 6-well plates in complete DMEM. After 48 h, the culture media was refreshed and 1 μM staurosporine (Sigma Aldrich) was added for 1, 2, or 3 h. The cells were washed in PBS and labeled with Annexin V PE and 7-AAD for 15 min at room temperature (BD Biosciences). Autophagy markers were evaluated in DC2.4^WT^ or DC2.4^*Dab2*−*/*−^ cells after incubation with 200 nM rapamycin (Sigma Aldrich) or 100 nM bafilomycin for 6 h in complete DMEM at 37°C. Cells incubated under the same conditions to induce cell death or autophagy were analyzed by Western blotting.

### Statistics

Statistical analyses were performed with Prism 7 (GraphPad Software; Figures S2A–D). One-way ANOVA with Tukey *post-hoc* test was used to compare the means. A *p* ≤ 0.05 was considered significant.

## Results

### Dab2 Is Predominantly Expressed in CD11b^+^ Intestinal Dendritic Cells and Is Downregulated During Experimental Colitis

**Figure 1:**
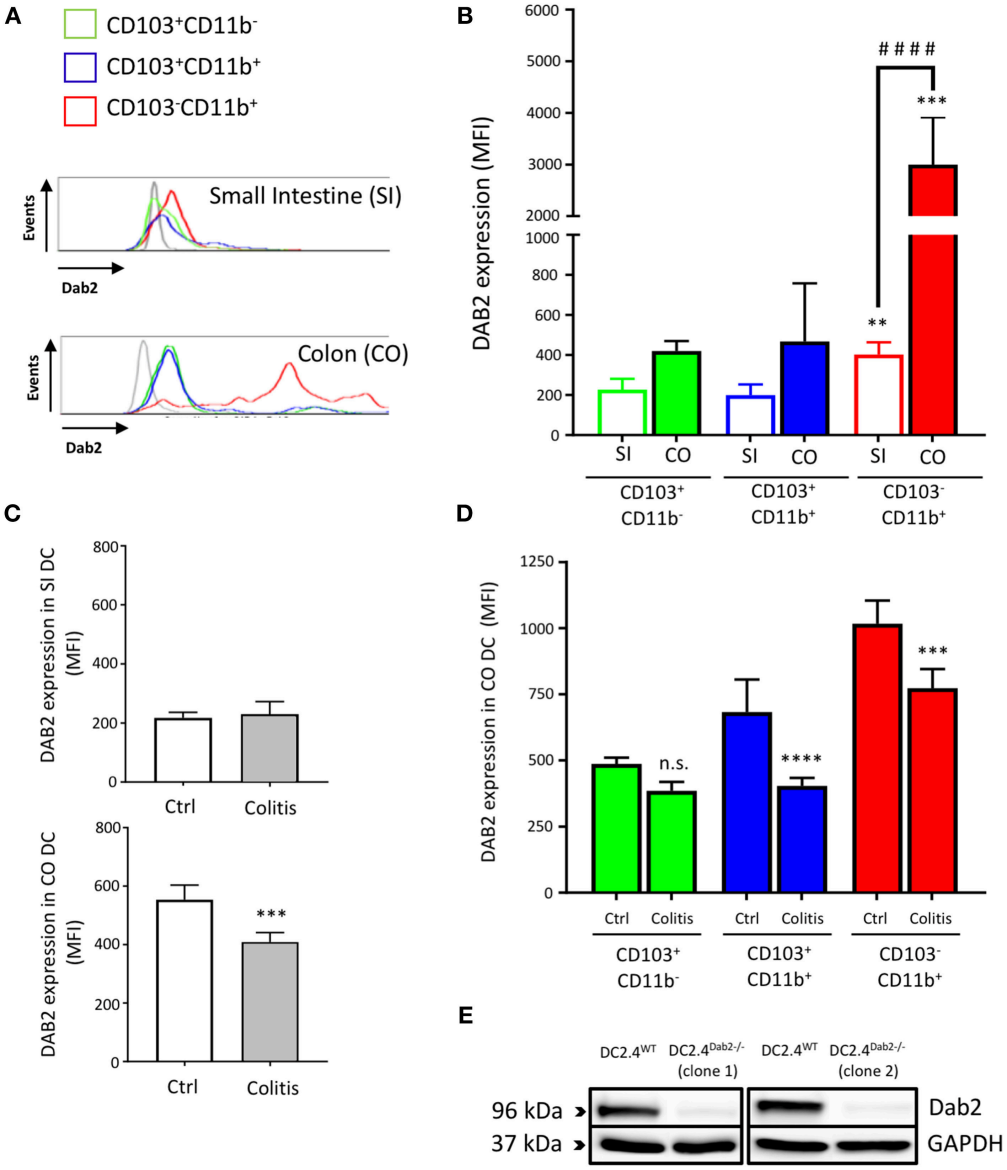
Dab2 is highly expressed in colonic lamina propria CD103^−^CD11b^+^ DCs and downregulated during murine colitis. **(A,B)** Flow cytometric analysis of DAB2 expression in small intestinal (SI) and colonic (CO) mucosal DCs of healthy WT C57BL/6J mice; **p < 0.01 and ***p < 0.005 when compared to other; ^*####*^*p* < 0.001 when comparing the indicated groups DC subsets from the same tissue. **(C,D)** Flow cytometric analysis of DAB2 expression in the SI and CO DC in Rag2^−/−^ mice after T cell transfer (Colitis) or injection with PBS (Ctrl) (***p < 0.005 and ****p < 0.001 Colitis vs. Ctrl); n.s., not significant. DCs were analyzed by flow cytometry after staining with viability dye eFluor 506, anti-F480, CD11c, CD11b, CD103, and anti-DAB2 mAbs. DC are depicted as live F480^−^CD11c^+^ and stratified by CD11b and CD103 expression. Data represent DAB2 expression as mean fluorescence intensity (MFI) of two independent experiments combined (*n* = 5 mice/experiment). **(E)** Western blotting analysis of DAB2 expression (96 kDa) in WT (DC2.4^WT^) and Dab2^−/−^ (clones 1 and 2; DC2.4^*Dab2*−*/*−^*)* DC2.4 cells after transfection with *Dab2* CRISPR-CAS9 and clonal selection.

We isolated leukocytes from small intestine and colon lamina propria of C57BL/6J mice and analyzed DAB2 expression on the main subsets of intestinal dendritic cells gated on live CD11c^+^F4/80^−^ cell populations: CD11^+^CD103^−^, CD11^+^CD103^+^, and CD11^−^CD103^+^ (Figure S1). CD103^−^CD11b^+^ DCs expressed the highest levels of DAB2 in both localities when compared to CD103^+^CD11b^+^ and CD103^+^CD11b^−^ DCs (Figure 1). DAB2 expression was higher in colonic CD103^−^CD11b^+^ DCs compared to the same subset in the small intestine (Figures 1A,B), which may indicate an important role of DAB2 in colonic DCs that have to handle higher microbial exposure. We evaluated DAB2 expression in the lamina propria DCs in adoptive naïve T cell transfer colitis. As anticipated, T cell transfer resulted in a significant weight loss and increased inflammatory cytokines in the colon (Figure S2). DAB2 protein was significantly downregulated in colonic DCs from the inflamed tissue when compared to DCs from control mice, particularly among the CD11b^+^ cells (Figures 1C,D). There was no significant change in DAB2 expression in small intestinal DCs from T-cell transferred mice (Figure 1C).

### Dab2-Deficient DCs Exacerbate Experimental Colitis

**Figure 2:**
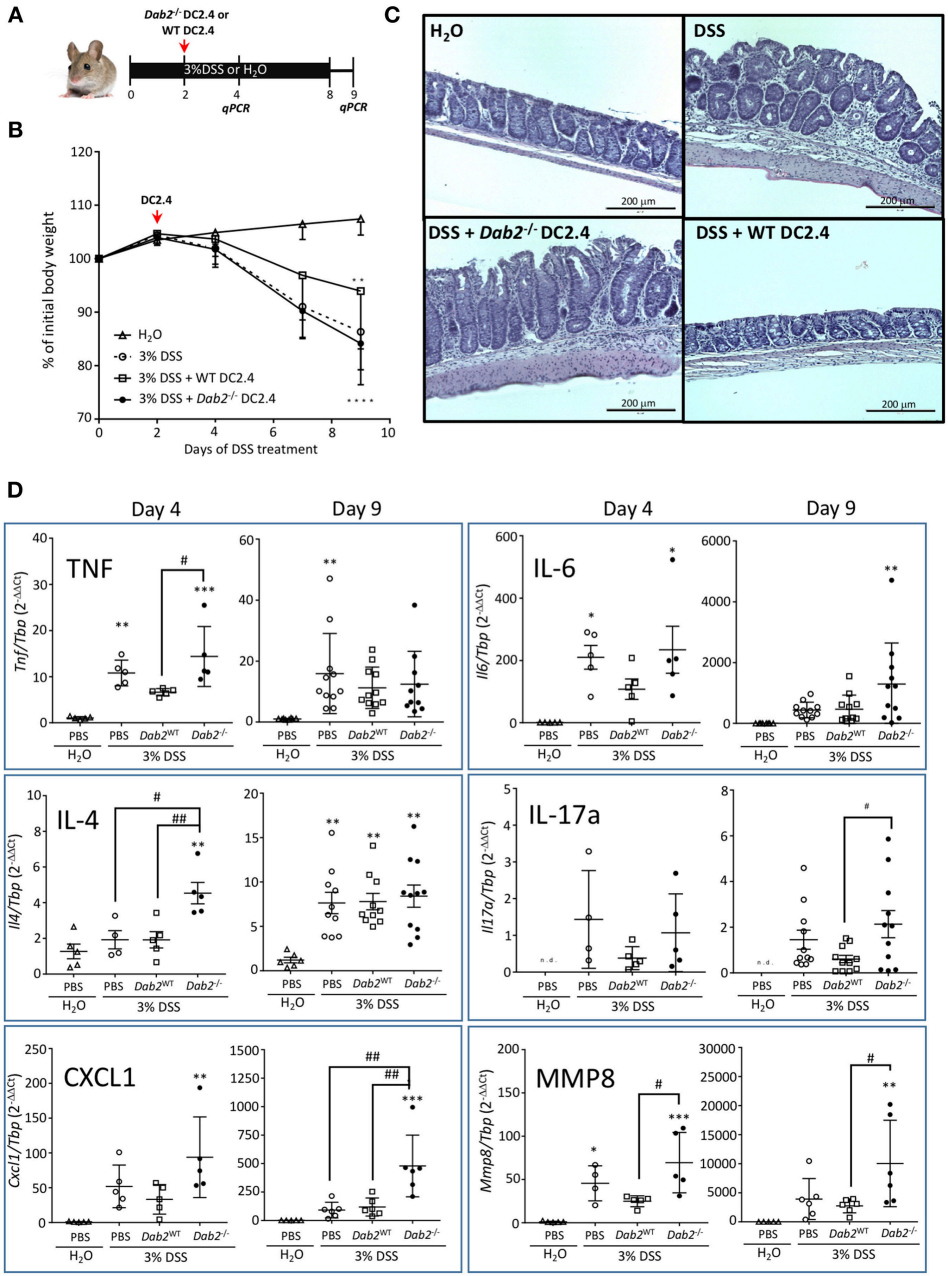
Pro-inflammatory effect of Dab2-deficient DCs during DSS colitis. **(A)** C57BL/6J mice received 3% DSS in their drinking water or regular water for 8 days. On day 2, mice were intraperitoneally injected with 8.5 × 10^5^ DC 2.4 WT or Dab2^−/−^ cells. Mice were switched to regular water on day 8. Two cohorts of mice were euthanized on days 4 or 9 after DSS treatment. **(B)** Relative body weight change; *n* = 8–11 **p < 0.01 and ****p < 0.001 DSS vs. H_2_O in mice treated with DC2.4WT and DC2.4^*Dab2*−/−^ cells, respectively. **(C)** Representative H&E staining of the distal colons from each experimental group (day 9). Bar = 200 μm. **(D)** Colonic gene expression of selected inflammatory mediators in control and DSS-treated mice by qRT-PCR on days 4 and 9 after DSS treatment. *n* = 5–11 (*p < 0.05, **p < 0.01 and ***p < 0.005 when compared to untreated mice or ^#^*p* < 0.05 and ^*##*^*p* < 0.01 in pairs indicated by brackets.

To further determine the contribution of Dab2 downregulation in DCs to intestinal inflammation, we ablated expression of *Dab2* in DC2.4 cells (murine immortalized dendritic cells) using the CRISPR-CAS9 system, resulting in 2 clones (#1 and #2) of Dab2^−/−^ cells (Figure 1E). A 1:1 mix of the two clones was used for further experiments. DC2.4^WT^ or DC2.4^*Dab2*−*/*−^ syngeneic DCs were injected into C57BL/6J mice on day 2 after treatment with 3% DSS. The extent of inflammation was evaluated on days 4 and 9 after the beginning of DSS treatment (Figure 2A). More pronounced colonic inflammation was observed in mice injected with DC2.4^*Dab2*−*/*−^ cells when compared to mice injected with DC2.4^WT^ cells, with the latter partially protecting mice from weight loss (Figure 2B), tissue inflammation (Figure 2C), and colonic cytokine production (Figure 2D). Mice injected with DC2.4^*Dab2*−*/*−^ cells demonstrated higher mucosal expression of *Tnf*α and *Il4* on day 4, and higher *Il6* and *Il17a* on day 9 following DSS treatment; additionally, *Cxcl1* (a neutrophilic chemoattractant) and mucosal *Mmp8* (a marker gene for neutrophil infiltration) increased at both timepoints (Figure 2D). Moreover, a transient significant increase in *Il1*β and *Il12a* was observed on day 4 but was no longer detected 9 days after induction (Figure S3). Therefore, the loss of DAB2 in mucosal dendritic cells may be an early contributor to the intestinal inflammation during experimental colitis.

### Dab2 Expression in BMDCs Is Downregulated After Activation of Toll-Like Receptors

**Figure 3:**
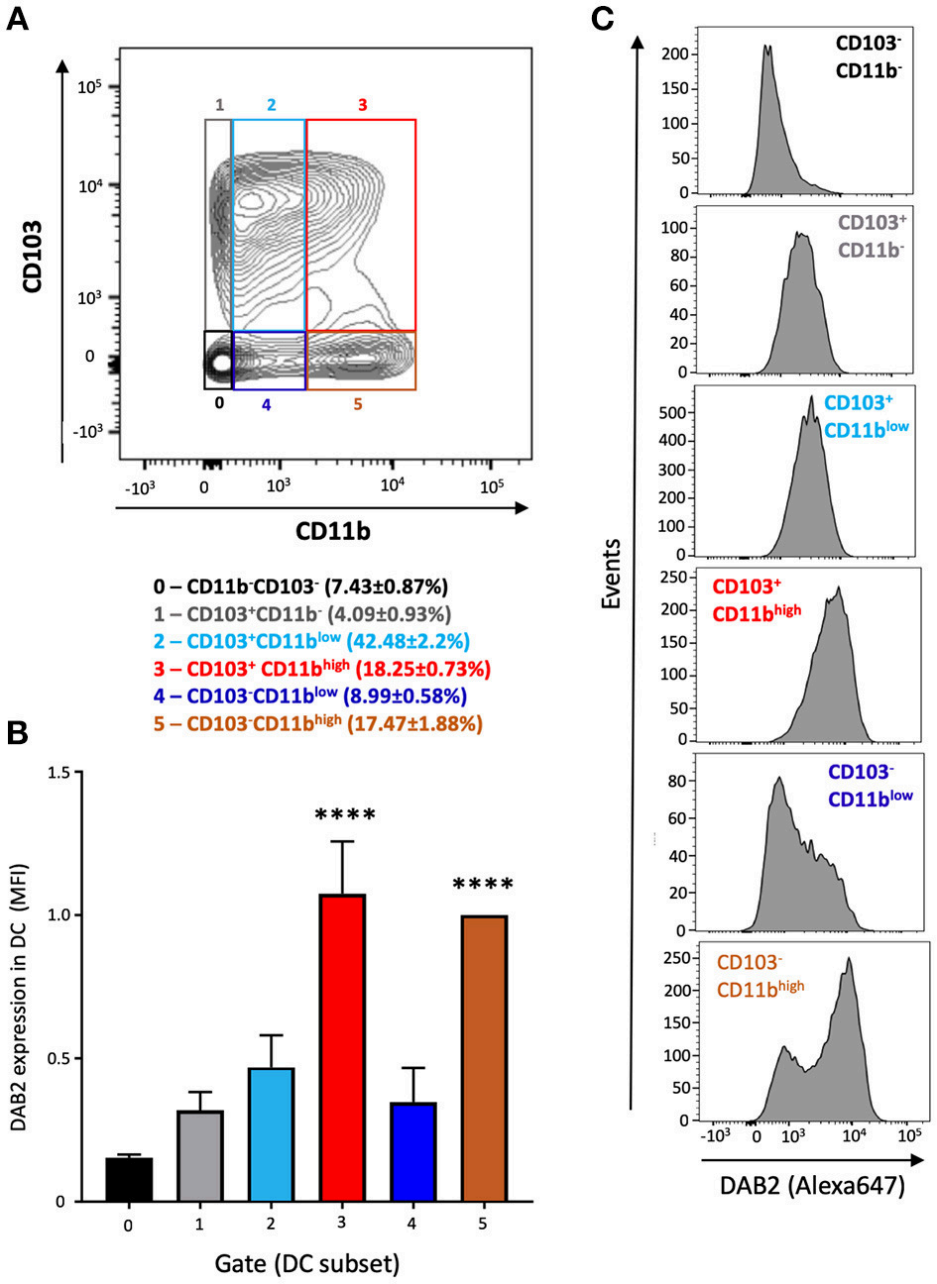
DAB2 is expressed in bone marrow derived dendritic cells from C57L/6J mice and correlates with CD11b expression. Bone-marrow derived dendritic cells (BMDCs) were differentiated from C57BL/6J mice with a combination of 200 ng/mL FLT3L for 9 days and 20 ng/mL GM-CSF for the last 2 days and analyzed by flow cytometry after staining with viability dye eFluor 506, anti-F480, B220, CD11c, CD11b, CD103, and anti-DAB2 mAbs. Conventional DC are depicted as live F480^−^B220^−^CD11c^+^ and stratified by CD11b and CD103 expression. **(A)** Relative abundance of BMDC phenotypes based on the expression of CD11b (neg, low, or high) and CD103 (neg, pos) (gates 0–5). **(B)** DAB2 expression in BMDCs is expressed as mean fluorescence intensity (MFI) in each gate. **(C)** Representative histograms of DAB2 expression in the populations gates as in **(A)**. Data represent mean values of three independent experiments combined (*n* = 3 mice/experiment ****p < 0.001 when compared to the gates/populations 0, 1, 2, and 4).

**Figure 4:**
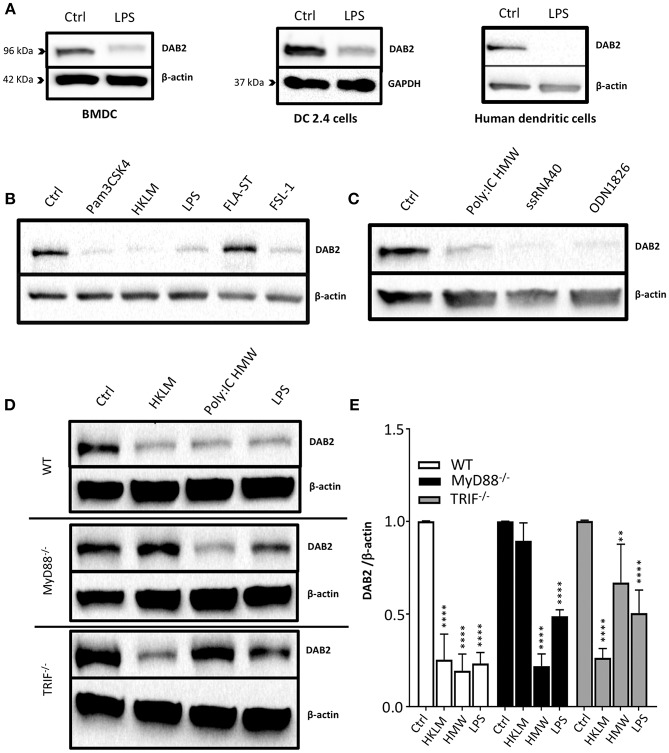
DAB2 is downregulated in human and murine dendritic cells after Toll-like receptor activation through MyD88 and TRIF pathways. **(A)** Bone-marrow derived dendritic cells (BMDC) differentiated from C57BL/6J mice, DC2.4 cells, and human monocytes-derived dendritic cells were incubated with 100 ng/mL LPS, and DAB2 expression was quantified using western blotting**. (B)** DAB2 expression in BMDCs treated with agonist for extracellular TLR or **(C)** agonist for intracellular TLR. **(D,E)** DAB2 expression after treatment with HKLM, HMW, or LPS in BMDC differentiated from WT, MyD88^−/−^, and or TRIF^−/−^ C57BL/6 mice. Bar graphs represent mean values of three independent experiments combined (*n* = 3 mice, **p < 0.01, ****p < 0.001 treatment vs. Ctrl within the same genetic background).

To address the mechanisms of DAB 2 downregulation during colitis, we generated BMDCs *in vitro* using FLT3L and GM-CSF, a protocol capable of inducing a more heterogeneous mix of DCs based on their CD103 and CD11b expression (35) (Figure 3A). DAB2 was expressed in all of the different subsets of BMDCs, and consistent with the primary mucosal DC, CD11b^+^ BMDCs expressed the highest levels of DAB2, regardless CD103 expression (Figures 3A–C). To test the potential role of TLR4 signaling in DAB2 downregulation, DAB2 expression was investigated in BMDC, DC2.4 cells or human-monocyte derived DCs incubated overnight with LPS. DAB2 expression was significantly reduced in the three types of DCs under these conditions (Figure 4A). To investigate whether this effect was specific to TLR4 pathway, BMDCs were incubated with TLR1-9 agonists and DAB2 expression was evaluated by Western blotting. DAB2 expression in BMDCs was reduced after exposure to both extracellular (Figure 4B) and intracellular TLR ligands (Figure 4C), with the exception of TLR5-agonist, flagellin (FLA); most likely because BMDCs differentiated under our protocol do not express detectable TLR5 (data not shown). TLR signaling requires one or two of the major adapter proteins, MyD88 and TRIF, and both have been demonstrated as required for antigen presenting cells to instruct T cell differentiation. We observed that downregulation of DAB2 by TLR agonists involves both MyD88 and TRIF adaptor proteins. DAB2 downregulation was abolished in BMDCs from MyD88^−/−^ mice treated with HKLM (TLR2 agonist that signals exclusively through MyD88) and in BMDCs from TRIF^−/−^ mice treated with poly I:C HMW (TLR3 agonist that signals exclusively via TRIF) (Figures 4D,E). As expected, DAB2 downregulation by LPS was only partially restored in BMDCs from either TRIF^−/−^ or MyD88^−/−^ mice, since TLR4 utilizes both adapters (Figures 4D,E). Taken together, these observations indicate that TLR activation ubiquitously affects DCs to reduce DAB2 expression in a MyD88/TRIF dependent-manner.

### DAB2 Protein and mRNA Are Downregulated by TLR4 Stimulation in a Biphasic Fashion

**Figure 5:**
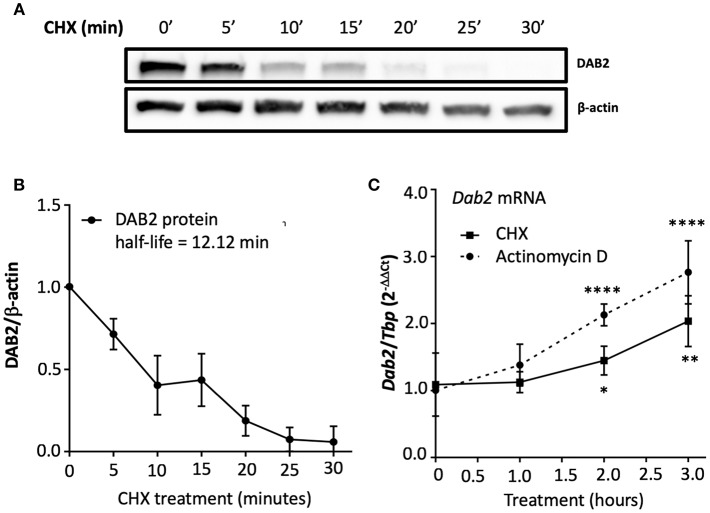
DAB2 expressed in dendritic cells has an unstable protein but a stable transcript. **(A,B)** BMDCs were incubated with 5 μg/mL cycloheximide (CHX) for up to 30 min and DAB2 expression was evaluated by western blotting. **(C)** Time course analysis of *Dab2* mRNA after treatment with 10 μg/mL actinomycin D or 5 μg/mL CHX (experiment was limited to 3 h to avoid toxicity and cell death). *Dab2* and *Tbp* gene expression was quantified using qRT-PCR. *p < 0.05, **p < 0.01, ****p < 0.001 effect of the treatment when compared to time 0. Data represent mean values from at least two independent experiments combined (*n* = 3 mice/experiment).

**Figure 6:**
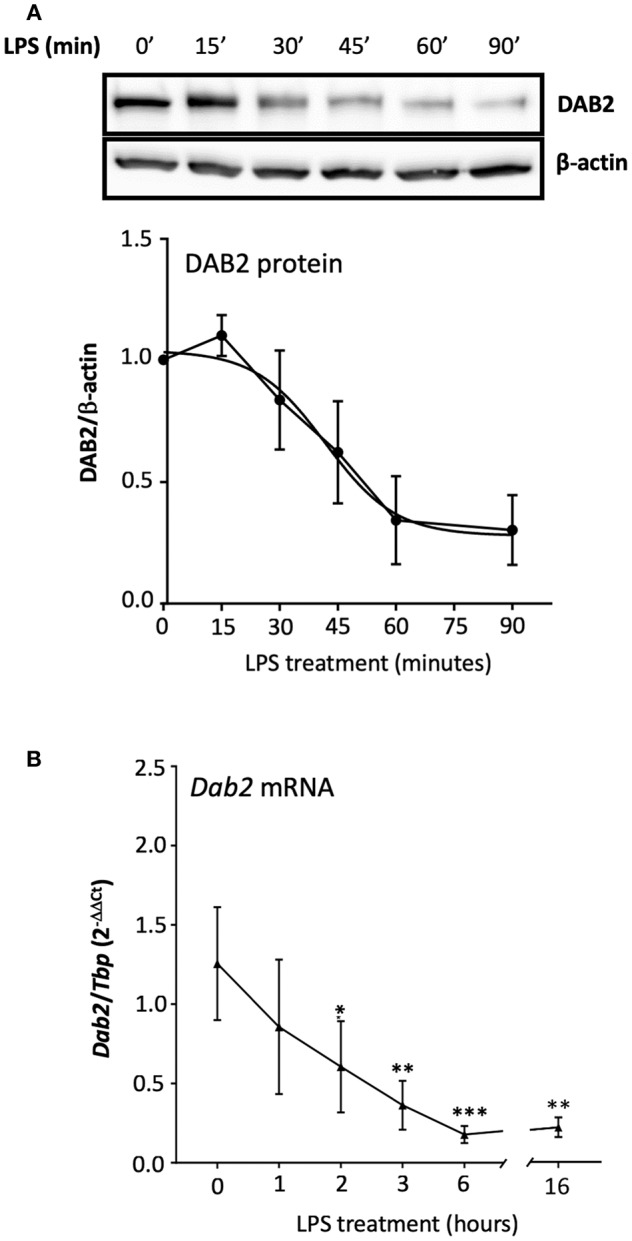
TLR4 activation represses both DAB2 protein and gene expression with different dynamics.**(A)** BMDCs were incubated with 100 ng/mL LPS for up to 90 min and DAB2 protein expression was evaluated by Western blotting. **(B)** *Dab2* mRNA expression was evaluated after incubation with 100 ng/mL LPS for up to 16 h. *Dab2* and *Tbp* (as an internal control) gene expression was quantified using qRT-PCR. *p < 0.05, **p < 0.01, ***p < 0.005 effect of LPS compared to time 0. Data represent mean values of at least two independent experiments combined (*n* = 3 mice each).

It has been shown that the half-life of DAB2 protein in macrophages is relatively long (8 h in RAW 264.7 cells) (29). To study DAB2 stability in dendritic cells, we incubated BMDCs from C57BL/6J mice with cycloheximide (CHX), a translational inhibitor (Figure 5A), or actinomycin D (Figure 5B), a transcriptional inhibitor, and DAB2 protein and mRNA were analyzed over time. We found that DAB2 expression in DCs was particularly unstable, with a half-life of ~12 min (Figures 5A,B). We observed a steady increase in *Dab2* mRNA in cycloheximide-treated DCs, and even more so with actinomycin D-treated cells (Figure 5C), suggesting that *Dab2* transcript is more stable, and that at baseline, the mRNA level is negatively regulated by a protein and/or miRNA with a relatively short lifespan. We next examined DAB2 protein and mRNA kinetics in BMDCs after LPS exposure. LPS led to a rapid decline of DAB2 protein (Figure 6A), with a maximum reduction at 60 min of incubation, before any significant changes in *Dab2* mRNA occurred (Figure 6B). Reduced DAB2 protein expression was sustained for at least 24 h after LPS exposure (data not shown). *Dab2* mRNA was gradually reduced (by >60% compared to control cells) in LPS-treaded BMDCs, with the first statistically significant time point occurring at 2 h and continuing a downward trend for at least 16 h after treatment (Figure 6B). These data demonstrate that DCs respond to TLR4 stimulation with a rapid downregulation of the unstable DAB2 protein, which is then subjected to long term suppression via a transcription/translation-dependent mechanism.

### Decline of DAB2 Protein in Dendritic Cells After TLR4 Activation Does Not Involve Lysosomal or Proteasomal Degradation

**Figure 7:**
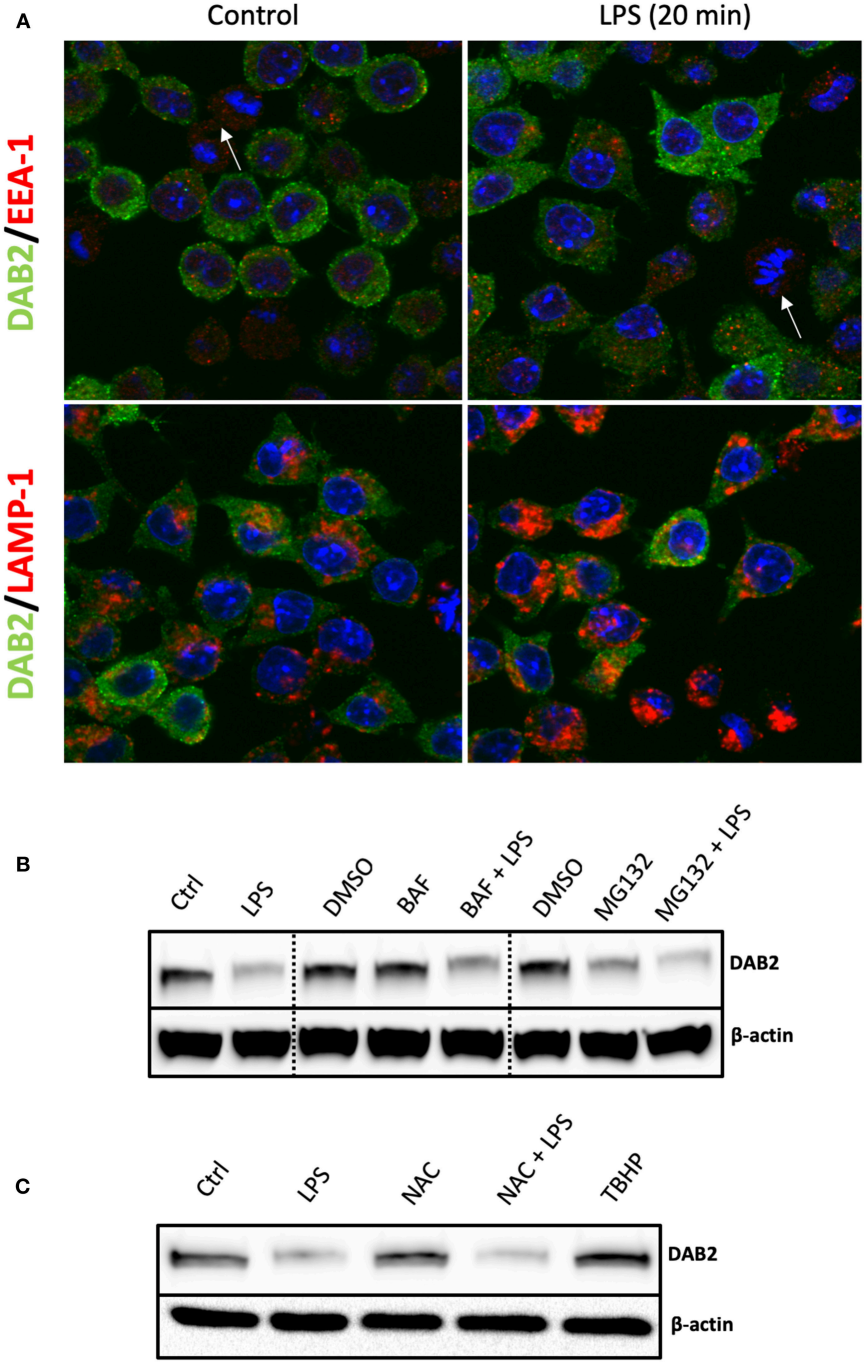
DAB2 protein downregulation after TLR activation does not require proteasome or lysosomal degradation. **(A)** DAB2 (green) and EEA-1 or LAMP-1 (red) were immunolabeled in DC2.4 cells exposed to control medium or to 100 ng/mL LPS for 20 min and images were acquired using confocal microscopy. Arrows indicate cells undergoing mitosis. There was no co-localization of DAB2 with early endosomal (EEA-1) or lysosomal (LAMP-1) positive compartments. **(B)** DAB2 expression was assessed by western blotting in cells pre-treated with 2 μM MG132, 100 nM Bafilocymin A1 or DMSO (vehicle) in culture media for 30 min before 1 h treatment with 100 ng/mL LPS. **(C)** DAB2 expression was assessed by Western blotting in control cells, cells treated for 1 h with LPS, treated with 2 mM n-acetyl-cysteine (NAC) for 1 h, cells pre-treated 15 min with NAC before 1 h LPS treatment, or cells treated with 55 μM tert-butyl-hydroperoxide (TBHP) for 1 h. Data represents results from least three independent experiments.

In order to define the mechanism of Dab2 downregulation in DCs after TLR activation, we evaluated the two major pathways that mediate protein degradation in eukaryotic cells, lysosomal proteolysis and the ubiquitin-proteasome pathway. Immunofluorescence analysis demonstrated a pronounced decline in DAB2 in DC2.4 cells undergoing mitosis (Figure 7A), which could be part of the mechanism involved in the temporary interruption of clathrin mediated endocytosis during cell division (36). However, we observed no co-localization of DAB2 with the endosomal or lysosomal markers (EEA-1 and LAMP, respectively) at baseline or after 20 min of LPS treatment (Figure 7A). Moreover, DAB2 downregulation by LPS did not involve either of the two pathways, since the treatment with MG132, a proteasome inhibitor, or bafilomycin A1, pH gradient-collapsing lysosomal V-ATPase inhibitor, were not able to prevent the rapid decline of DAB2 protein in LPS-treated DCs (Figure 7B). Both drugs were shown to be effective under our experimental conditions as observed when quantifying LC3A/B II and p-IκBα (Ser^32^) accumulation after lysosomal and proteasome inhibition (Figure S4). A pH-independent lysosomal proteolysis regulated by NADPH oxidase Nox2 has been described in macrophages by Rybicka et al. (37). TLR-mediated human DC maturation was associated with increased Nox2 and enhanced superoxide release, both of which are required for lysophagosomal-mediated microbial killing (38). However, inhibition of ROS production by N-acetylcysteine (NAC) pre-treatment before TLR4 activation did not restore DAB2 expression, and baseline DAB2 expression was not affected by tert-Butyl hydroproxide (TBHP), an oxidative stress inducer (Figure 7C). These finding confirm no involvement of the lysosomal pathway in the immediate repression of DAB2 after TLR4 activation. Inhibition of TLR downstream signaling using inhibitors of NF-kB, p38, or JNK MAPK also did not restore DAB2 expression in LPS-treated BMDCs (data not shown), collectively demonstrating a rapid, non-canonical mechanism of DAB2 control in activated dendritic cells.

### Loss of DAB2 Leads to Decreased Phagocytosis in Dendritic Cells

**Figure 8:**
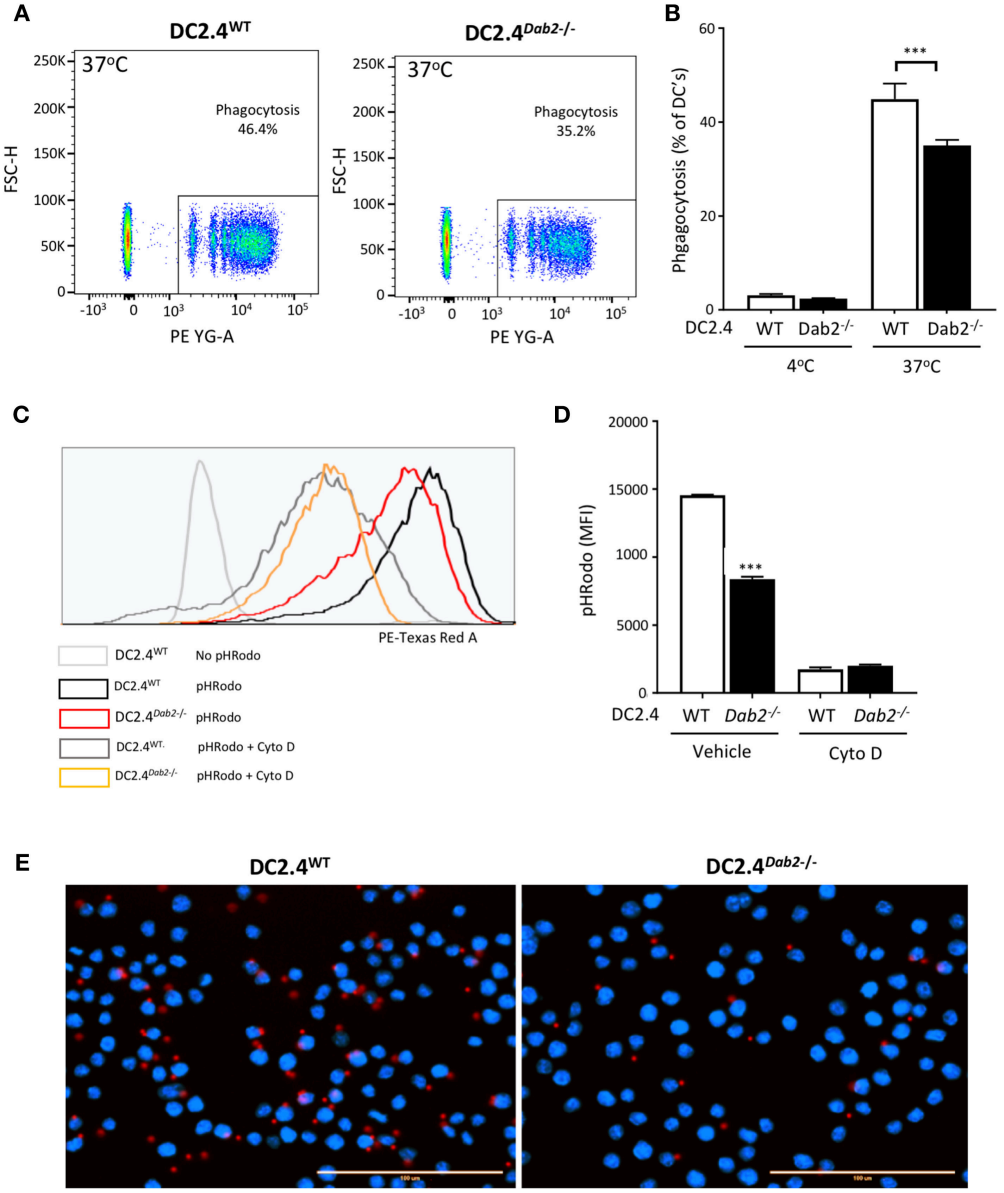
DAB2 regulates DC phagocytosis. **(A,B)** Internalization of IgG-coated latex beads cells was evaluated after incubation of DC2.4^WT^ or DC2.4^*Dab2*−*/*−^ cells at a ratio of 25 beads/cell for 1 h at 37°C. The percentage of DCs that internalized red beads was analyzed by flow cytometry. Data represent mean values of *n* = 3, ***p < 0.005 DC2.4^WT^ vs. DC2.4^*Dab2*−*/*−^ cells. **(C,D)** Histogram showing mean fluorescence intensity (MFI) obtained from DC2.4^WT^ vs. DC2.4^*Dab2*−*/*−^ cells incubated with pHrodo Red *E. coli* BioParticles for 1 h in the presence of vehicle or 1 μM cytochalasin D **(D)** ***p < 0.005 DC2.4^WT^ vs. DC2.4^*Dab2*−*/*−^ cells. **(E)** Representative epifluorescent images of DC2.4^WT^ vs. DC2.4^*Dab2*−*/*−^ cells after 1 h of phagocytosis of the BioParticles at 37°C. Nuclei are depicted in blue (DAPI) and beads in red. Bar = 100 μm. Data representative of at least three independent experiments.

Active phagocytosis, characteristic of immature DCs, is reduced after microbial antigen exposure, while the cells gain the migratory ability to home to local peripheral lymph nodes. To determine whether DAB2 downregulation after TLR activation (a maturation signal) could contribute to the reduced phagocytosis observed in mature DCs, we tested phagocytosis of red fluorescent IgG-coated beads or *E. coli* bioparticle pHrodo™ by DC2.4^WT^ or DC2.4^*Dab2*−*/*−^ cells. We observed that Dab2 depletion in DC2.4 cells indeed led to a decreased uptake of IgG-coated beads (Figures 8A,B) and of the *E. coli* bioparticles (Figures 8C,D), when compared to WT cells. The reduced phagocytosis of the red fluorescent IgG-coated beads in DC2.4^*Dab2*−*/*−^ cells was confirmed by fluorescence microscopy (Figure 8E). Cytochalasin D, a potent inhibitor of actin polymerization, or incubation at 4°C, were used as negative controls for phagocytosis.

### Dab2 Expression Regulates *in vitro* Activation and Cytokine Production in Dendritic Cells

**Figure 9:**
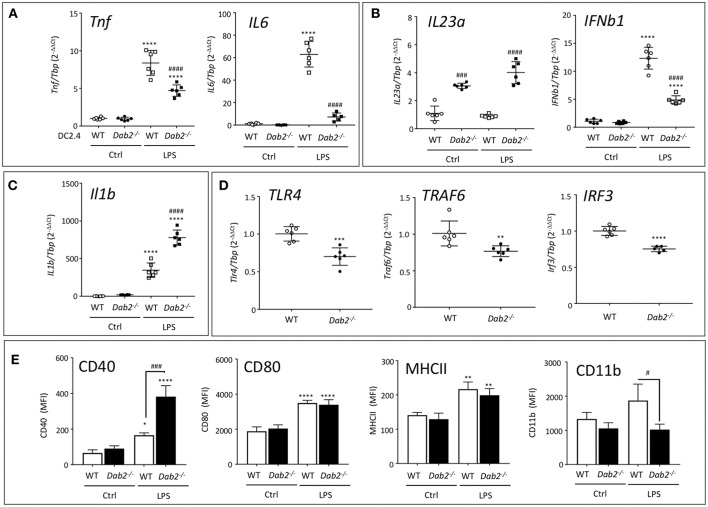
DAB2 expression modulates DC response to TLR activation. mRNA expression of **(A)** *Tnf* and *Il6*, **(B)** *Il23a* and *Ifn*β*1*, **(C)** *Il1*β, and **(D)** *Tlr4, Traf6*, and *Irf3* 6 h after exposure of DC2.4^WT^ or DC2.4^*Dab2*−*/*−^ cells to 100 ng/mL LPS, evaluated by qRT-PCR with *Tbp* used as an internal control. **(E)** Expression of cell surface DC activation markers CD40, CD80, MHCII, and CD11b in DC2.4^WT^ vs. DC2.4^*Dab2*−*/*−^ cells treated with vehicle (Ctrl) or 100 ng/mL LPS for 24 h was evaluated by flow cytometry and expressed as mean fluorescence intensity (MFI). Data represent mean values of *n* = 3–6. (*p < 0.05, **p < 0.01, ***p < 0.05 and ****p < 0.001 Ctrl vs. LPS for DC2.4^WT^ or DC2.4^*Dab2*−*/*−^ cells. ^#^*p* < 0.05 and ^*###*^*p* < 0.005, ^*####*^*p* < 0.001 when DC2.4^WT^ vs. DC2.4^*Dab2*−*/*−^ cells in the same treatment groups).

To address the direct impact of DAB2 downregulation induced by TLR agonists on DC activation, we treated DC2.4^WT^ or DC2.4^*Dab2*−*/*−^ with PBS or LPS for 6 h and analyzed the expression of selected cytokines and DC activation markers using RT-qPCR and flow cytometry. The expression of *Tnf*α and *Il6* (Myd88-dependent cytokines) were not different between DC2.4^WT^ or DC2.4^*Dab2*−*/*−^ cells at baseline, but loss of *Dab2* led to a significant decrease in LPS-stimulated *Tnf*α and *Il6* mRNA (Figure 9A). *IL23a* mRNA was increased in *Dab2*-deficient cells at baseline, and the loss of *Dab2* potentiated the effect of LPS on *IL23a* expression, but reduced *Ifnb1* response (both TRIF-dependent cytokines; Figure 9B). Similar to *Il23a*, the expression of *Il1*β (MyD88/TRIF dependent cytokine) was significantly higher in LPS-treated or DC2.4^*Dab2*−*/*−^ cells compared to LPS-treated DC2.4^WT^ DCs (Figure 9C).

DAB2 has been described interacting with TRAF6 in human embryonic kidney cells (29), and with TRIF in macrophages (30), impacting these branches of the intracellular signaling triggered by TLR. At baseline, when compared to DC2.4^WT^ cells, DC2.4^*Dab2*−*/*−^ DCs had significantly decreased expression of *Tlr4, Traf6*, and *Irf3* (Figure 9D), but not of *Myd88* or *Trif* (Figure S5).

In flow cytometric analysis of the DC activation markers MHCII and CD80 expression was not affected by *Dab2* status, although CD40 expression was dramatically upregulated by LPS in DC2.4^*Dab2*−*/*−^ DCs compared to DC2.4^WT^ cells (Figure 9E). Interestingly, CD11b induction by LPS was abolished in DC2.4^*Dab2*−*/*−^ cells (Figure 9E). The histograms corresponding to CD40 and CD11b expression in DC2.4^WT^ and DC2.4^*Dab2*−*/*−^ cells after treatment with LPS are shown in Figure S6. These observations suggest a degree of specificity in the control of cell surface marker expression. Indeed, we observed no alteration in the cell surface expression of chemokine receptors, CXCR4, CCR9, or CCR7 between LPS-activated DC2.4^WT^ and DC2.4^*Dab2*−*/*−^ cells (Figure S7).

Since TGFβ signaling is known for controlling the DC activation state, and Dab2 was implicated in the control of the canonical TGFβ signaling pathway in fibroblasts (22), we tested if TGFβ signaling is also affected in *Dab2*-deficient DCs. However, we found that TGFβ-stimulated SMAD2 phosphorylation was not affected by *Dab2* knockout (Figure S8). Thus, DAB2, while it does not control the canonical TGFβ signaling, is an important modulator of DC phenotype and it differentially and selectively alters the expression of cytokines and cell surface activation markers produced in response to TLR4 activation.

### Dab2 Expression Affects Autophagy and Protects Dendritic Cells From Cell Death

**Figure 10:**
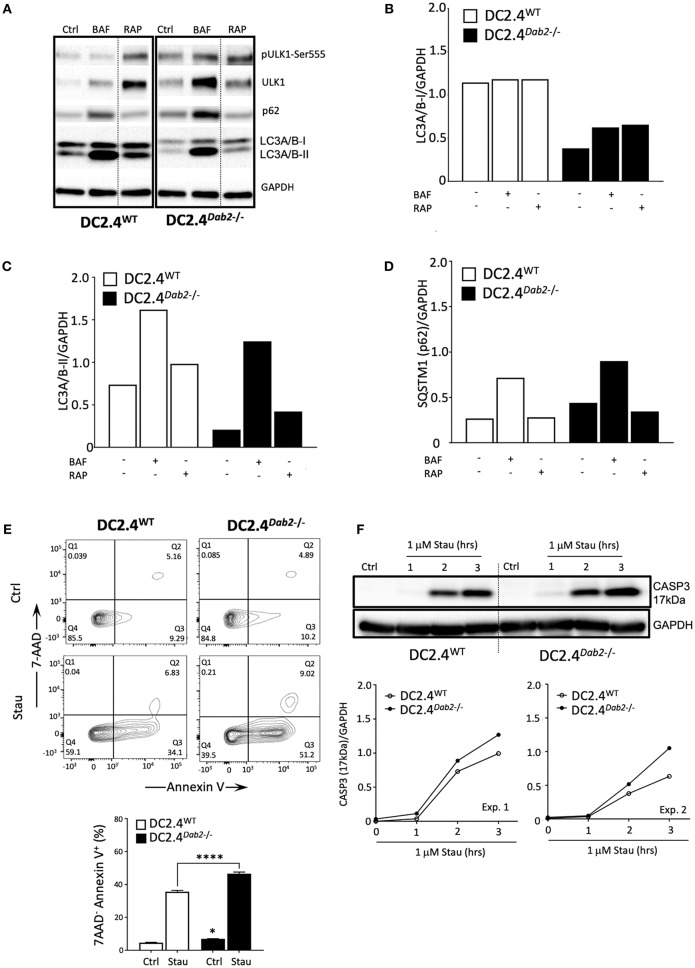
DAB2 modulates autophagy and protects dendritic cells from apoptosis. **(A)** DC2.4^WT^ and DC2.4^*Dab2*−*/*−^ cells were left untreated or treated either with 100 nM bafilomycin A1 (BAF) or 200 nM rapamycin (RAP) for 6 h and the expression of autophagy markers, p-UKL-1, UKL-1, p62, LC3A/B I-II, was evaluated by Western blotting. **(B)** Summary of relative changes in LC3A/B-I expression. **(C)** Summary of relative changes in LC3A/B-II expression. **(D)** Summary of relative changes in SQSTM1**/**p62 expression. **(E)** Cell death in DC2.4^WT^ and DC2.4^*Dab2*−*/*−^ cells treated with vehicle or 1 μM staurosporine for 3 h was accessed by flow cytometry to identify AnnexinV^+^7-AAD^−^ apoptotic cells (*p < 0.05 Ctrl DC2.4^WT^ vs. Ctrl DC2.4^*Dab2*−*/*−^ cells; ****p < 0.001 Stau DC2.4^WT^ vs. Stau DC2.4^*Dab2*−*/*−^ cells). **(F)** Caspase-3 activation after incubation with 1 μM staurosporine for 1, 2, and 3 h was accessed using Western blot. The samples compared were separated in the same gel. Due to inter-experimental variation in the time-course of caspase 3 activation, two out of three independent experiments performed are depicted.

Macroautophagy (herein referred to as autophagy) is a catabolic process that targets intracellular components (organelles, protein aggregates, infectious organisms, etc.), contained within a *de novo*-formed, double membraned vesicle called an autophagosome, to the lysosome to be degraded. The resulting energy and biomolecules obtained following autophagosome/lysosomal fusion assist in the maintenance of cellular homeostasis and serve an energy source in response to cellular stress. In DCs, autophagy has a negative impact on the immunogenic maturation and is downregulated after activation with LPS (39). It is known that Il-1β and IL23a mRNA and protein expression are regulated by autophagy in antigen presenting cells (APC) and inhibition of autophagy favors their expression and secretion in response to TLR3 and TLR4 agonists (40, 41). Given the observation that *Dab2* expression impacts DC maturation and phagocytosis, we investigated the role of *Dab2* in DC autophagy. To determine whether *Dab2* knockout or downregulation, as observed in DCs after TLR activation, affects autophagy in DCs we investigated the expression of the specific autophagy markers pULK1-Ser555 (associated with autophagy induction), LC3A/B-I (the cytoplasmic-borne LC3 isoform), LC3A/B-II (the lapidated LC3 isoform incorporated into autophagosomes) and Sequestosome 1/p62 (SQSTM1/p62—a protein that targets protein aggregates to autophagosomes) in DC2.4^WT^ or DC2.4^*Dab2*−*/*−^ DCs. We observed that both LC3A/B-I and -II proteins were constitutively reduced in DC2.4^*Dab2*−*/*−^ cells (Figures 10A–C). Additionally, we observed a modestly increased accumulation of SQSTM1/p62, an autophagy substrate, in DC2.4^*Dab2*−*/*−^ cells, indicating reduced autophagic activity in the absence of DAB2 (Figure 10D). Furthermore, the levels of the total and phosphorylated UKL-1, an autophagy initiator, were increased in DC2.4^*Dab2*−*/*−^ cells, possibly indicating a compensatory mechanism to upregulate autophagy; however, the phosphorylation of ULK1 at Ser^555^ was reduced in DC2.4^*Dab2*−*/*−^ (pULK1-Ser555/ULK1 = 1.07) when compared to DC2.4^WT^ cells at baseline (pULK1-Ser^555^/ULK1 = 1.41), indicating a reduction in the basal pathway activation relative to DC2.4^WT^. Bafilomycin A1, an inhibitor of lysosomal acidification and as a result autophagosomal fusion, led to the accumulation of autophagosomes as demonstrated by a robust increase in LC3A/B-II in both DC2.4^WT^ and DC2.4^*Dab2*−*/*−^ cells, indicating that *Dab2* deficiency did not hamper autophagic flux (the rate of autophagic degradation) i.e., the autophagic machinery remains intact and functional in DC2.4^*Dab2*−*/*−^ cells (Figures 10A–C). Finally, treatment with rapamycin, an autophagy inducer, failed to bring the levels of LC3A/B-II and SQSTM1/p62 in DC2.4^*Dab2*−*/*−^ DCs to those observed in DC2.4^WT^ cells (Figures 10A–C). These data, taken together, indicate a reduction, but not a blockade, in autophagic activity in DC2.4^*Dab2*−*/*−^ cells.

As autophagic and apoptotic programs appear to inhibit each other (42), and apoptosis participates in the DC life cycle and elimination after maturation (8), we investigated if *Dab2* deletion could impact DC apoptosis. To test that, DC2.4^WT^ and DC2.4^*Dab2*−*/*−^ DCs were treated with staurosporine, an apoptosis inducer, for up to 3 h and apoptosis was detected by 7AAD/Annexin V labeling and flow cytometry and by Western blot analysis of the cleaved caspase-3 (17 kDa) expression. As compared to DC2.4^WT^ DCs, DC2.4^*Dab2*−*/*−^ cells stimulated with staurosporine showed increased apoptosis (as detected by the frequency of 7AAD^−^AnnexinV^+^ DC) (Figure 10E). The analysis of the cleaved caspase 3 level confirmed those observations (Figure 10F). Taken together, these findings demonstrate that the DAB2 decline, as observed after TLR activation, can negatively regulate autophagy and favor apoptosis, both events associated with DC maturation.

## Discussion

Dendritic cells are differentially distributed in the gastrointestinal tract. Under homeostatic conditions, they continually sample the environment for antigens and present them to T cells, promoting the differentiation of FoxP3^+^ inducible regulatory T cells (iTreg) and supporting a modest number of mucosal IFNγ^+^ Th1, and IL-17^+^ Th17 T cells, without causing overt inflammation. In IBD, a dysfunctional intestinal epithelial barrier and microbial dysbiosis lead to an abnormal exposure of APCs, driving them toward an inflammatory phenotype capable of generating pathogenic Th1, Th2, or Th17 responses (11). However, the mechanisms that govern the phenotypic alterations in intestinal DCs after excessive microbial exposure and TLR activation are not fully understood. In this study we demonstrate that DAB2 contributes to the orchestrated immune response in DCs during intestinal homeostasis and inflammation. We observed that DAB2 is differentially expressed in the small intestinal and colonic DCs, with colonic CD103^−^CD11b^+^ DCs expressing the highest level of the protein. This cell population resides not only in the most heavily populated microbial environment but is also capable of orchestrating Th1 and Th17 immune responses in the gut. Thus, it became plausible that DAB2 may contribute to hypo-responsiveness of this DC population to commensal bacteria and their associated antigens. Moreover, we observed that during experimental colitis, DAB2 was downregulated in CD11b^+^ colonic DCs, regardless of CD103 expression, suggesting that decreased DAB2 expression may indeed be consequential during DC maturation. In support of this hypothesis, Dab2 repression in macrophages has been described to contribute to adipose and liver inflammation (29, 32).

To begin to understand the mechanisms of DAB2 downregulation in activated DCs, we showed that DAB2 expression in these cells was exquisitely sensitive to an array of TLR ligands, suggesting the importance of its downregulation during the immune response. Moreover, our data indicates a pathway allowing for rapid and sustained suppression of DAB2. We found DAB2 protein to be particularly unstable in DCs (ca. 12 min half-life), which permits rapid response when required. Indeed, the immediate drop in DAB2 protein levels following LPS treatment, but preceding a change in the mRNA levels, suggested protein degradation as an initial step. We excluded the proteasomal or pH-dependent and ROS-dependent lysosomal degradation as a mechanism, which implied a yet undefined non-canonical degradative pathway involved in regulating DAB2 protein stability in DCs. It was proposed that DAB2 sequesters clathrin from its interaction with TLR4. According to this hypothesis, TLR4 activation in macrophages leads to clathrin release from DAB2 and allows for the formation of clathrin domains near the cytoplasmic domain of TLR4 (30). Whether such a mechanism occurs in dendritic cells, and whether association with clathrin stabilizes DAB2 at the cytoplasmic membrane and protects it from degradation remains unknown.

The decline in *Dab2* mRNA represented the second phase of downregulation after LPS exposure. Epigenetic and transcriptional mechanisms in *Dab2* mRNA decline have been reported in cancer cells, including miRNA (43–46). The steady increase of *Dab2* mRNA in actinomycin-treated DCs suggested the presence of an inhibitory factor regulating a steady-state level of *Dab2* transcript. This could be consistent with either protein or miRNA, since cycloheximide (translation inhibitor) also resulted in a similar, though less pronounced, gradual increase in *Dab2* mRNA (Figure 5C). The precise mechanisms responsible for both DAB2 protein (in) stability and *Dab2* mRNA levels in DCs remain to be elucidated.

This complex mechanism allowing for the profound, rapid, and sustained inhibition of DAB2 expression suggests the importance of this process in DC biology. To test whether DAB2 expressed in DCs indeed controls their tolerogenic/immunogenic potential, we generated *Dab2*-deficient DC2.4 DCs using CRISPR/Cas9. Their adoptive transfer modulated the course of DSS colitis by enhancing the early (4 days) colonic mucosal expression of inflammatory cytokines *Tnf*α, *Il1*β, *Il4*, and *Il12a*, and the subsequent increase in *Il6* and *Il17a*. Collectively, these data strongly suggest that DAB2 is downregulated in the course of experimental colitis and that its loss modulates mucosal dendritic cell function and promotes local inflammatory responses. These observations are consistent with the results of Ahmed et al. (33) who showed that DAB2 knockdown in DCs enhanced production of Th1 and Th17 cytokines in DC-T cell co-culture, but will need to be further confirmed with a conditional DC-specific knockout of *Dab2 in vivo*.

Relatively little is known about the mechanisms that restrain activation pathways triggered by LPS and downregulate the inflammatory response downstream of TLR activation in DCs. A pattern of co-expression of DAB2 and CD11b in the lamina propria DCs may not just be a matter of DC lineage but may imply a functional relationship between the two proteins. Despite its role in leukocyte migration and recruitment to the inflamed tissue, integrin alpha-M (CD11b), highly expressed in APC, has been demonstrated to inhibit TLR-mediated responses by activating Syk and promoting MyD88 and TRIF degradation (47). Deficiency in CD11b polarizes differentiation of Th17 and exacerbates DSS induced colitis (48, 49). In our study, BMDCs differentiated with FLT3L/GM-CSF had a variable level of CD11b expression, which allowed us to identify a striking positive correlation between DAB2 and CD11b expression. Moreover, *Dab2*^−/−^ DCs expressed less CD11b when treated with LPS, suggesting that there is a mechanistic link between the two proteins. DAB2 has been demonstrated to regulate endocytosis of another class of integrin, integrin β1, by interaction through its phosphotyrosine-binding domain and its recruitment to the EH domain scaffold proteins present in clathrin-coated pits (26). DAB2 also plays a role in endocytic recycling. It is thus plausible that DAB2 could regulate CD11b trafficking and membrane recycling in a similar manner.

Based on our study with adoptive transfer of *Dab2*-deficient DCs and DSS colitis, we postulated that DAB2 downregulation would modulate DC function and their response to TLR stimulation. Somewhat similar observations were made in macrophages, where DAB2 reduced TRIF-dependent cytokine production at a steady-state, and TLR4 activation led to clathrin release from DAB2 to allow TRIF endocytosis (30). We showed that *in vitro*, loss of *Dab2* enhanced CD40, *Il1*β and *Il23a* expression in DC2.4 cells stimulated with LPS. IL-23 is a central player in intestinal inflammation, orchestrating an inflammatory cascade involving TNFα, IL-6, and IL-17, and is a potentially attractive target for pharmaceutical intervention (50, 51). Additionally, the enhanced CD40 expression in the *Dab2*-deficient DCs could also account for their higher proinflammatory potential. High CD40 expression by DCs was recently demonstrated as directly responsible for decreased frequency of tolerogenic CD103^+^ DCs and of the immunosuppressive RORγt^+^Helios^−^ iTreg cells, and consequently exacerbated inflammatory Th1/Th17 responses, microbial dysbiosis and fatal colitis (52). Interestingly, the increased *Il23a* and *Il1*β mRNA expression in *Dab2*-deficient DCs was accompanied by reduced expression of *Tnf*α, *Il6*, and *IFN*β*1*, as well as of *Tlr4, Traf6*, and *Irf3*, mediators known for the control of the latter three cytokines. The seemingly opposite effects of *Dab2* deficiency on the selected cytokines was intriguing and may be related to DC autophagy. Autophagy is known to dampen excessive inflammation in response to TLR stimulation and negatively modulates the innate immune system (53). Inhibition of autophagy augments the transcription of IL-1β and consequently IL-23a in human and mouse APC (41, 54), while having an opposite effect on TNFα (55). Consistent with this notion, *Dab2*-deficient DCs showed an apparent defect in autophagy, primarily related to constitutively decreased LC3A/B-I and -II protein expression but not to a defective LC3A/B-I to -II isoform conversion. Induction of autophagy with rapamycin did not increase the LC3A/B-II protein in *Dab2*^−/−^ DCs to the levels observed in WT DCs. Since impaired autophagy in DCs has been also shown to promote intestinal inflammation in mice (56), which may at least to some extent account for the exacerbating effect of *Dab2*-deficient DCs in DSS colitis.

Autophagy, an evolutionarily ancient process of lysosomal self-digestion, remains in a dynamic relationship with the co-regulated phagocytosis. Deletion of *Dab2* in DC2.4 DCs, which mimicked the effects of TLR stimulation, decreased phagocytosis, an event consistent with DC maturation and TLR-driven suppression of the actin-dependent micropinocytosis (57) and efferocytosis (58). These results are not, however, fully consistent with an earlier report by Ahmed et al. (33), which demonstrated that *Dab2* knockdown improved antigen uptake. The observed differences may be related to our use of DC2.4 cells [immortalized cells morphologically and functionally similar to BMDCs (34)] or BMDCs differentiated under a different protocol to result in population diversity more reminiscent of the gut mucosal DCs (35), while Ahmed et al. (33) used GM-CSF-derived bone marrow cells—a mix of DCs and macrophages that questionably represent *in vivo* populations (59–61).

Downregulating autophagy in any system interferes with a variety of cellular processes, including apoptosis. Indeed, *Dab2*^*Dab2*−*/*−^ DCs showed increased susceptibility to a pro-apoptotic stimulus staurosporine, a prototypical ATP-competitive kinase inhibitor, as well as to the inhibition of DNA synthesis with mitomycin C (data not shown). These data suggest that, in addition to the pro-inflammatory effects of DAB2 downregulation in activated DCs, perhaps in later stages of the DC life cycle, this process may also contribute to the resolution of the inflammatory response in otherwise healthy individuals. Indeed, DC apoptosis reduces the adaptive immune response by limiting the Ag availability to T cells, and environments with significant DC apoptosis can be immunosuppressive. On the other hand, in individuals with genetic predisposition to chronic inflammation, such as IBD, inhibition of DAB2 in APC may be one of the early cues for establishing tissue inflammation.

In conclusion, our observations provide the first description of DAB2 expression pattern in the intestinal and colonic DCs. We demonstrate that it is rapidly and profoundly reduced during DC maturation and that its loss affects their tolerogenic/inflammatory potential *in vivo* and *in vitro*, including their response to TLR4 stimulation, autophagy, and susceptibility to apoptosis. While future approaches will need to more precisely identify the molecular mechanisms involved in each of those events, it is plausible that DAB2 is not only a key player on DC biology, but it may represent an attractive target for pharmacological modulation to preserve its tolerogenic effects.

## Author Contributions

VF: study concept and design, acquisition of data, analysis and interpretation of data, drafting and revision of manuscript. DJ and MG: acquisition of data, analysis and interpretation of data, revision of manuscript. MM-K: acquisition of data, revision of manuscript. CH: acquisition of data, analysis and interpretation of data. CC: acquisition of data. JW: acquisition of data, analysis and interpretation of data, critical revision of the manuscript for important intellectual content, obtained funding. FG: study supervision, critical revision of the manuscript for important intellectual content, obtained funding; PK: study supervision, study concept and design, analysis and interpretation of data, drafting of the manuscript, critical revision of the manuscript for important intellectual content. statistical analysis, obtained funding.

### Conflict of Interest Statement

The authors declare that the research was conducted in the absence of any commercial or financial relationships that could be construed as a potential conflict of interest.
