# Supplementary material for: Rapid Downregulation of DAB2 by Toll-Like Receptor Activation Contributes to a Pro-Inflammatory Switch in Activated Dendritic Cells
Source: Front Immunol. 2019 Feb 27;10:304. doi: 10.3389/fimmu.2019.00304 (PMC6400992; doi:10.3389/fimmu.2019.00304)
Supplement: Supplementary file 1 [file Data_Sheet_1.PDF]

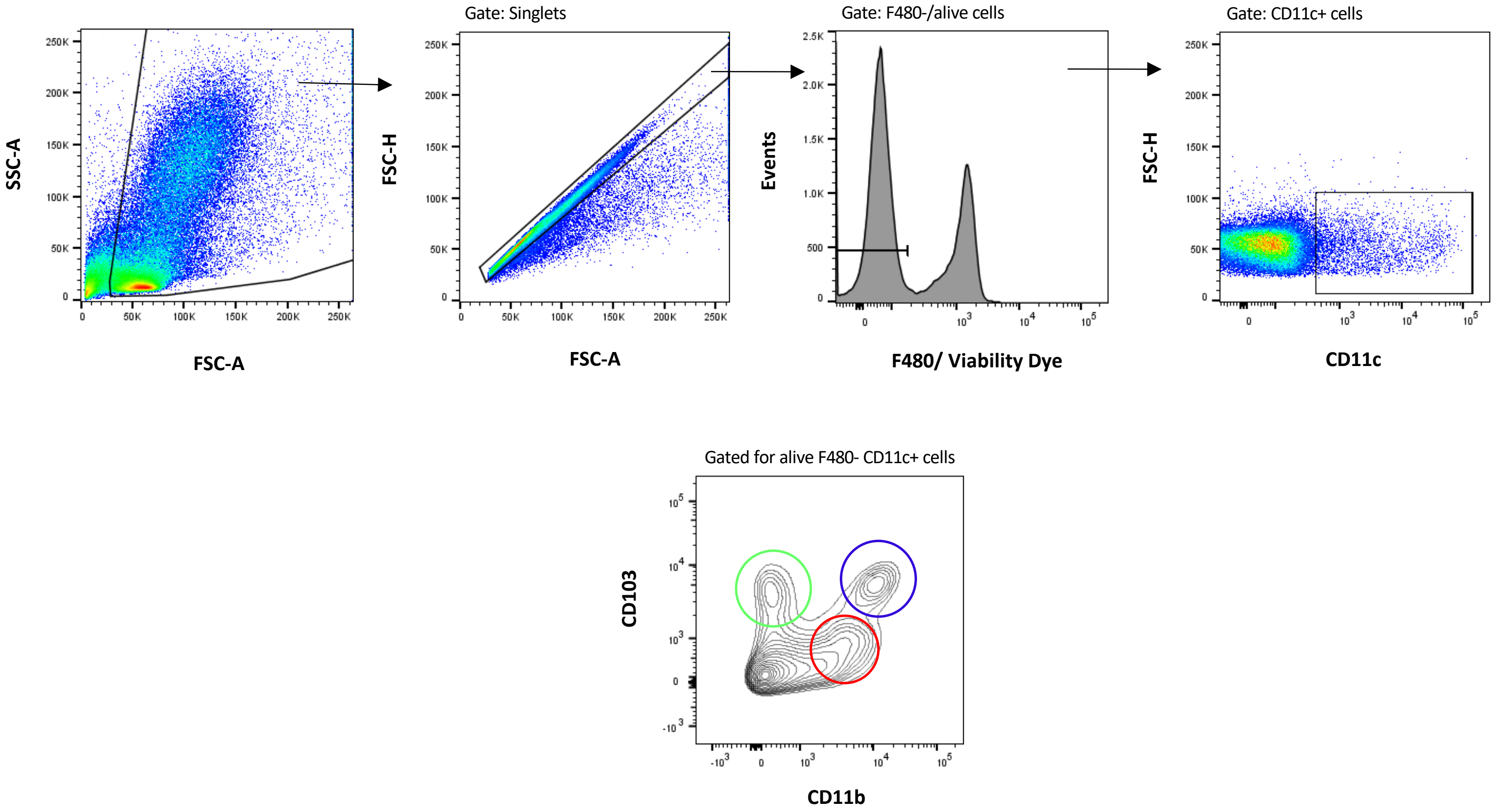

Fig. S1. Identifying lamina propria dendritic cells. Intestinal dendritic cells were gated as live F480^{-}CD11c^{+} cells followed by gating on CD11b^{-}CD103^{+}, CD11b^{-}CD103^{+} and CD11b^{-}CD103^{+} populations.

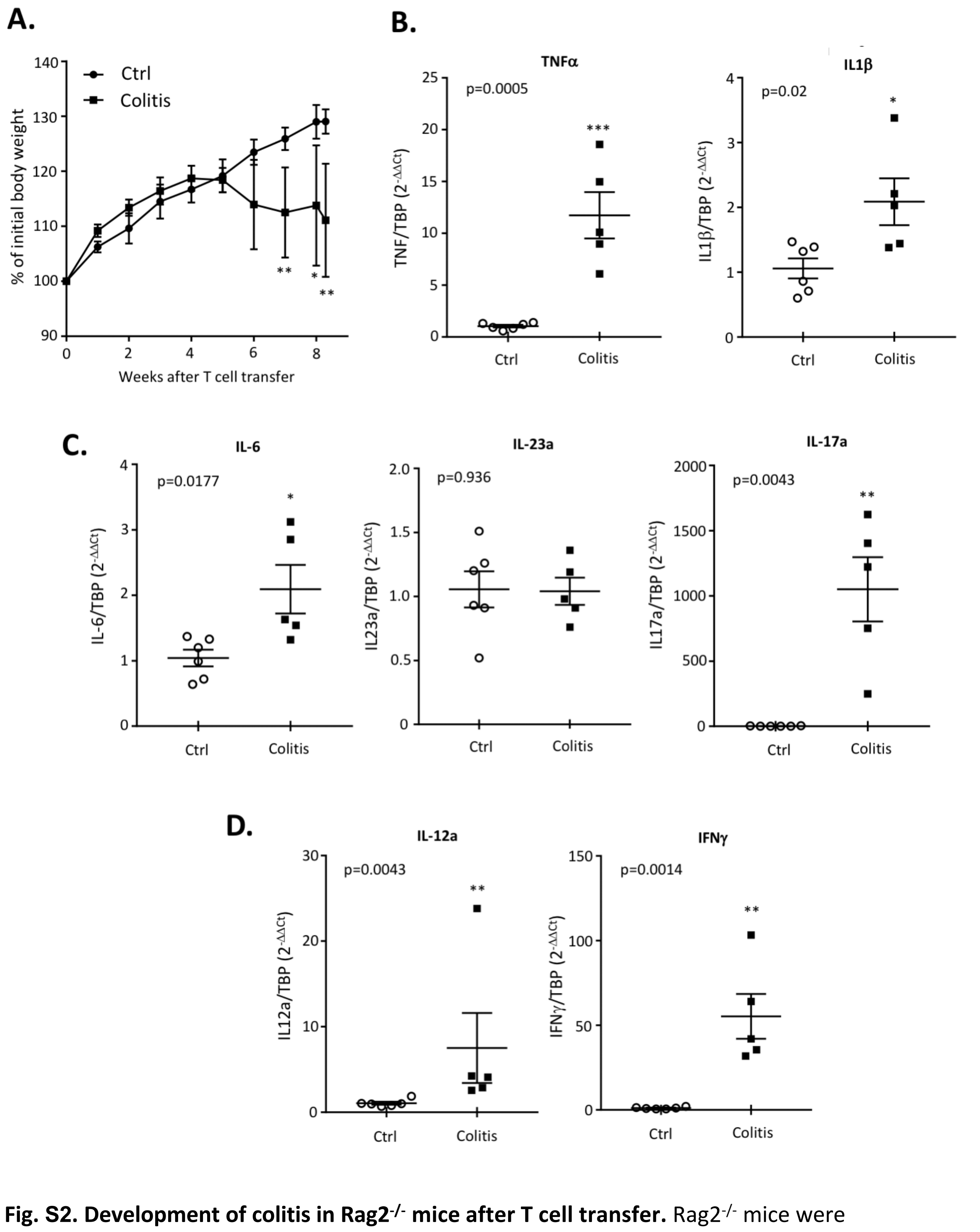

Fig. S2. Development of colitis in Rag2$^{-/-}$ mice after T cell transfer. Rag2$^{-/-}$ mice were intraperitoneally injected with 5.0×10^5 CD4$^+$CD45RB^{high} T cells or PBS on day 0 **(A)** Body weight was examined weekly for 58 days, when mice were euthanized for tissue collection. **(B)** Mucosal mRNA expression of *Tnf* and *Il1b*, **(C)** Mucosal mRNA expression of Th17 cytokines *Il6*, *Il23a* and *Il17a*. **(D)** Mucosal mRNA expression of Th1 cytokines *Il12a* and *Ifng* was measured using qRT-PCR. n=5 (* $p < 0.05$, ** $p < 0.01$ and *** $p < 0.005$ Ctrl. vs. Colitis).

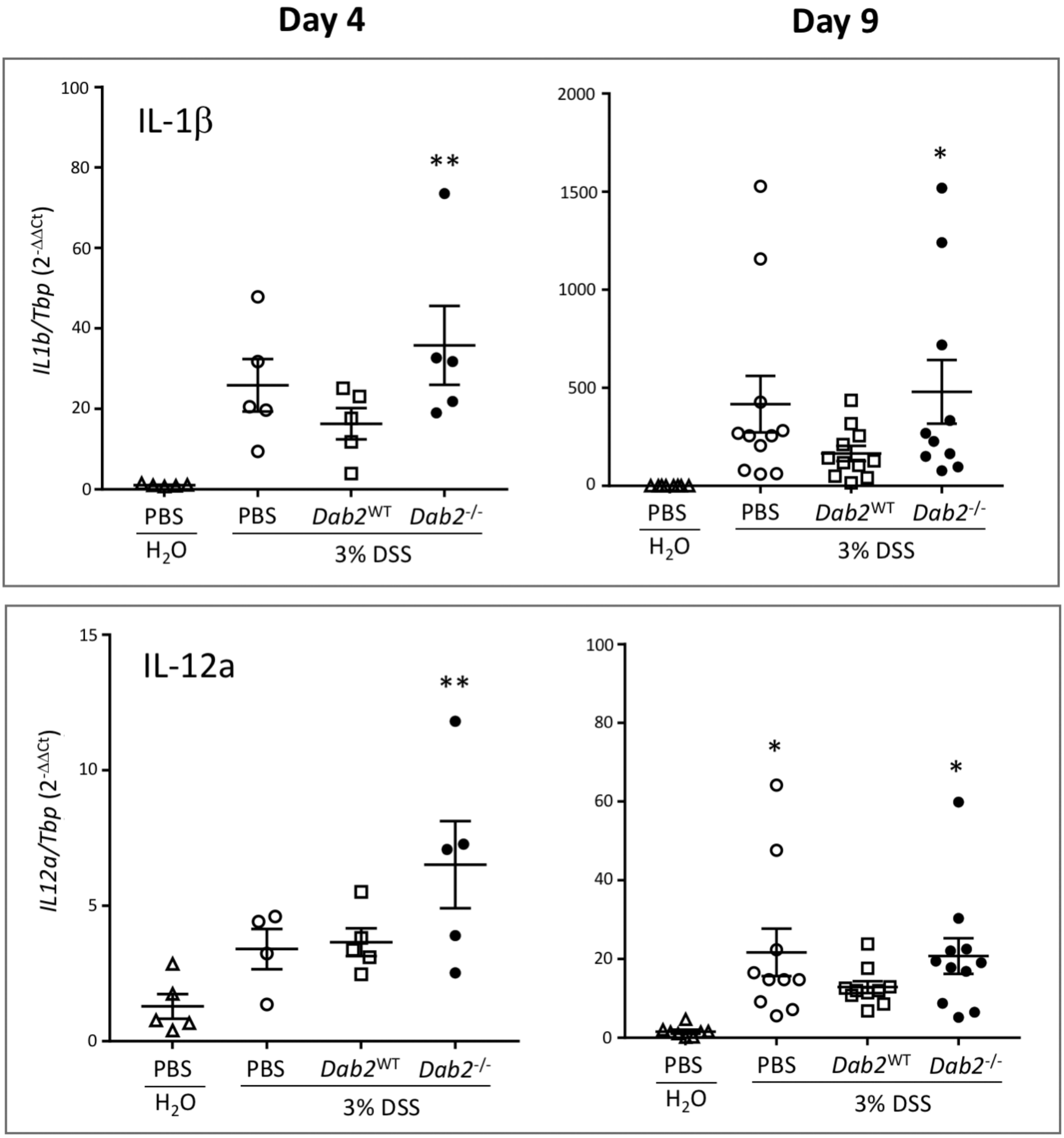

Fig. S3. Pro-inflammatory effect of Dab2-deficient DC during DSS colitis. C57BL/6J mice received 3% DSS in their drinking water or regular water for 8 days. On day 2, mice were intraperitoneally injected with 8.5×10^5 DC 2.4 WT or Dab2$^{-/-}$ cells. Mice were switched to regular water on day 8. Two cohorts of mice were euthanized on day 4 or day 9 after DSS treatment. Colonic expression of *IL1b* and *Il12a* mRNA in control and DSS-treated mice by qRT-PCR on day 4 and 9 after DSS treatment. n=5-11 (* $p < 0.05$, ** $p < 0.01$ when compared to untreated mice).

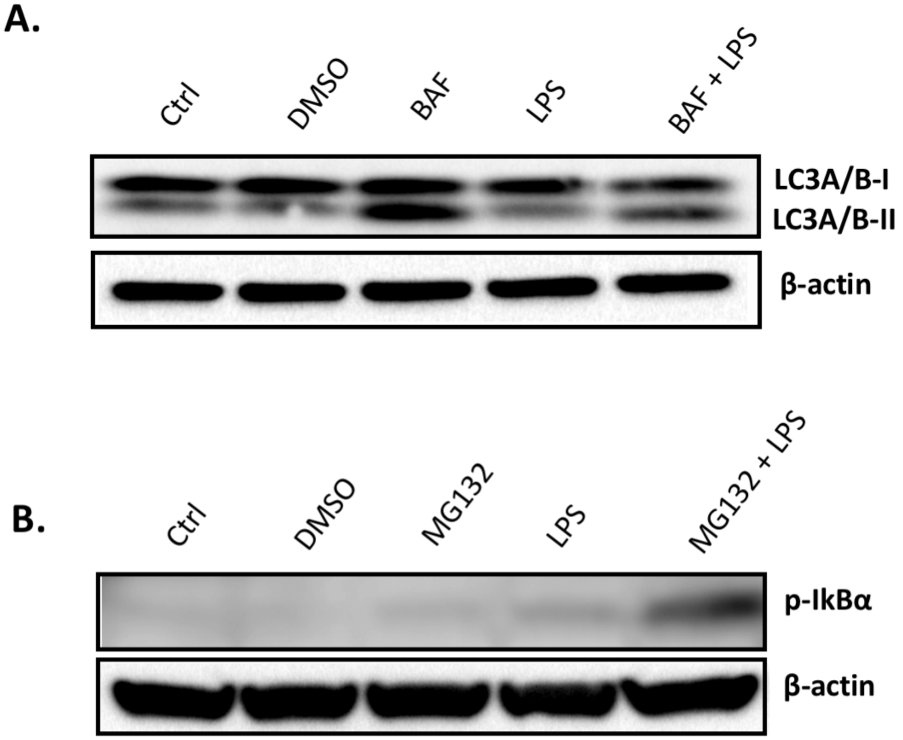

Fig. S4. Verification of proteasomal and lysosomal inhibition. (A) LC3A/B-I and LC3A/B-II protein expression was assessed by western blotting in BMDC pre-treated with 100 nM bafilocymin A1 for 30 min before 1-hour treatment with 100 ng/mL LPS. (**B**) p-IkBα was assessed by western blotting in cells pre-treated with 2 μM MG132 for 30 min before 1-hour treatment with 100 ng/mL LPS.

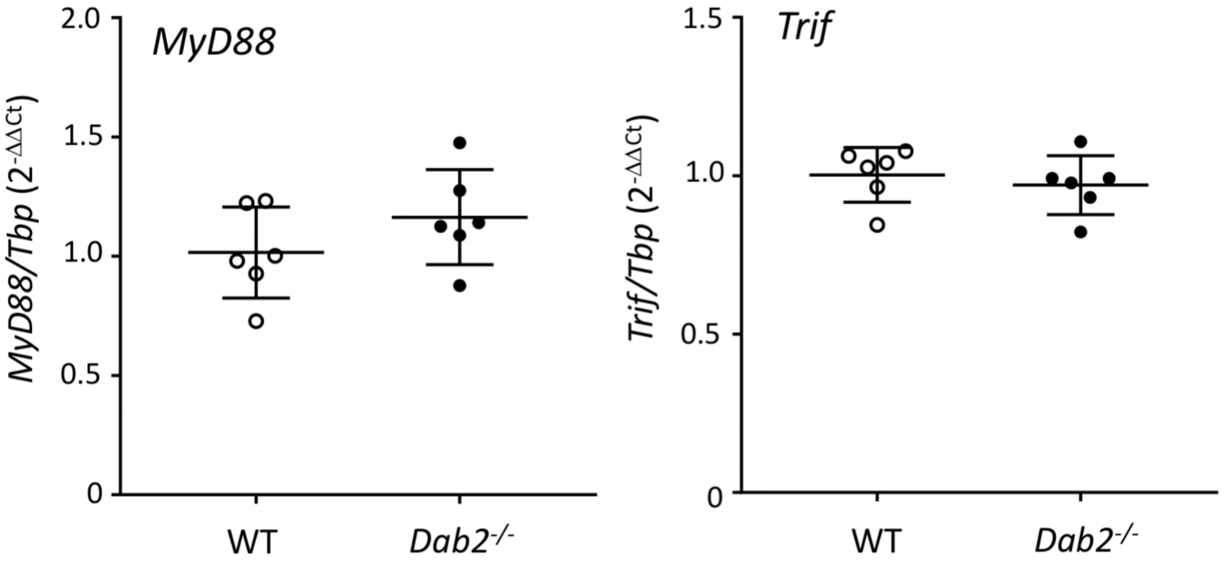

Fig. S5. *Dab2* knockout does not affect *Trif* or *MyD88* mRNA expression in dendritic cells. *MyD88* and *Trif* mRNA expression of was quantified in DC2.4^{WT} and DC2.4$^{Dab2-/-}$ cells using qRT-PCR with *Tbp* used as an internal control. Data represent mean values of n = 6.

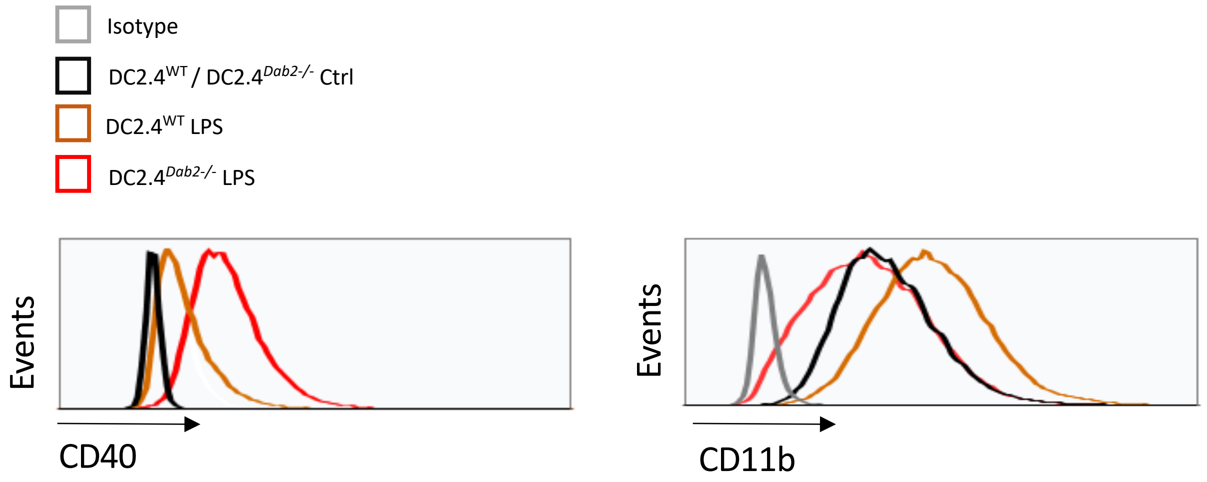

Fig. S6. Expression of CD40 and CD11b on DC2.4 cells. DC2.4^{WT} and DC2.4$^{Dab2-/-}$ cells were treated with vehicle (Ctrl) or 100 ng/mL LPS for 24 hours. Histogram of CD40 and CD11b expression in DC2.4 cells as evaluated by flow cytometry. Expression of CD40 and CD11b in DC2.4^{WT} / DC2.4$^{Dab2-/-}$ Ctrl cells is represented by black line in both panels.

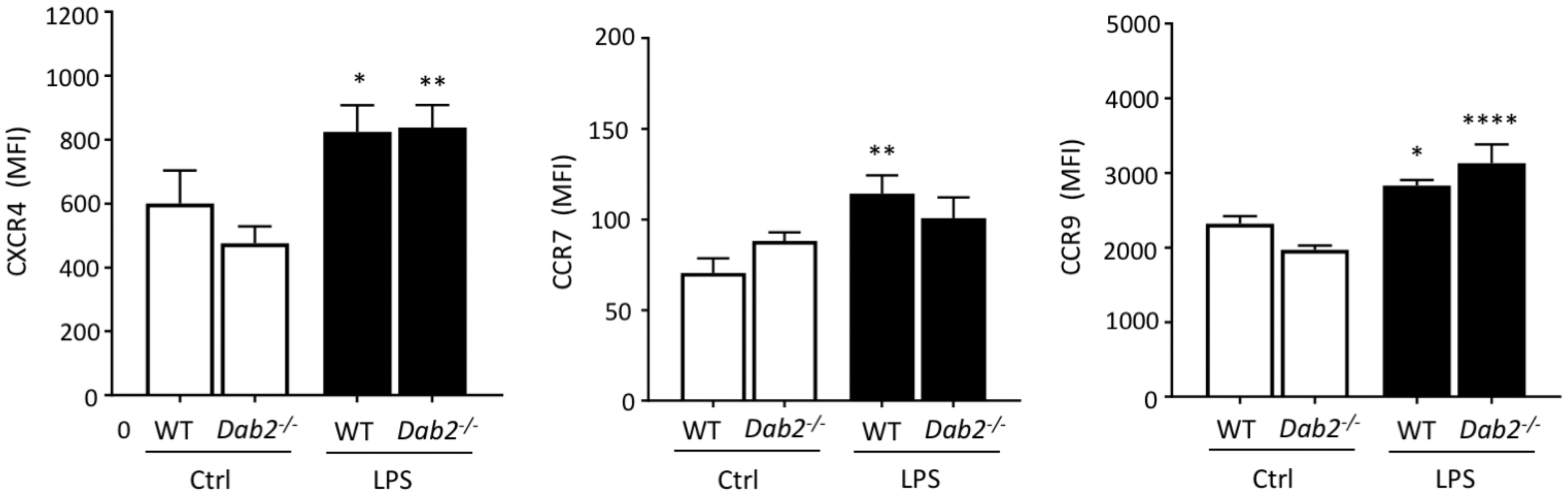

Fig. S7. *Dab2* expression does not affect the baseline or LPS-stimulated expression of chemokine receptors on the surface of dendritic cell. Expression of chemokine receptors CXCR4, CCR9 and CCR7 on the surface of DC2.4^{WT} and DC2.4$^{Dab2-/-}$ cells treated with 100 ng/mL LPS for 24 hours was evaluated by flow cytometry and expressed as mean fluorescence intensity (MFI). Data represent mean values of n = 3. (* $p < 0.05$, *** $p < 0.05$ and **** $p < 0.001$ when compared to the untreated cells of the same genotype).

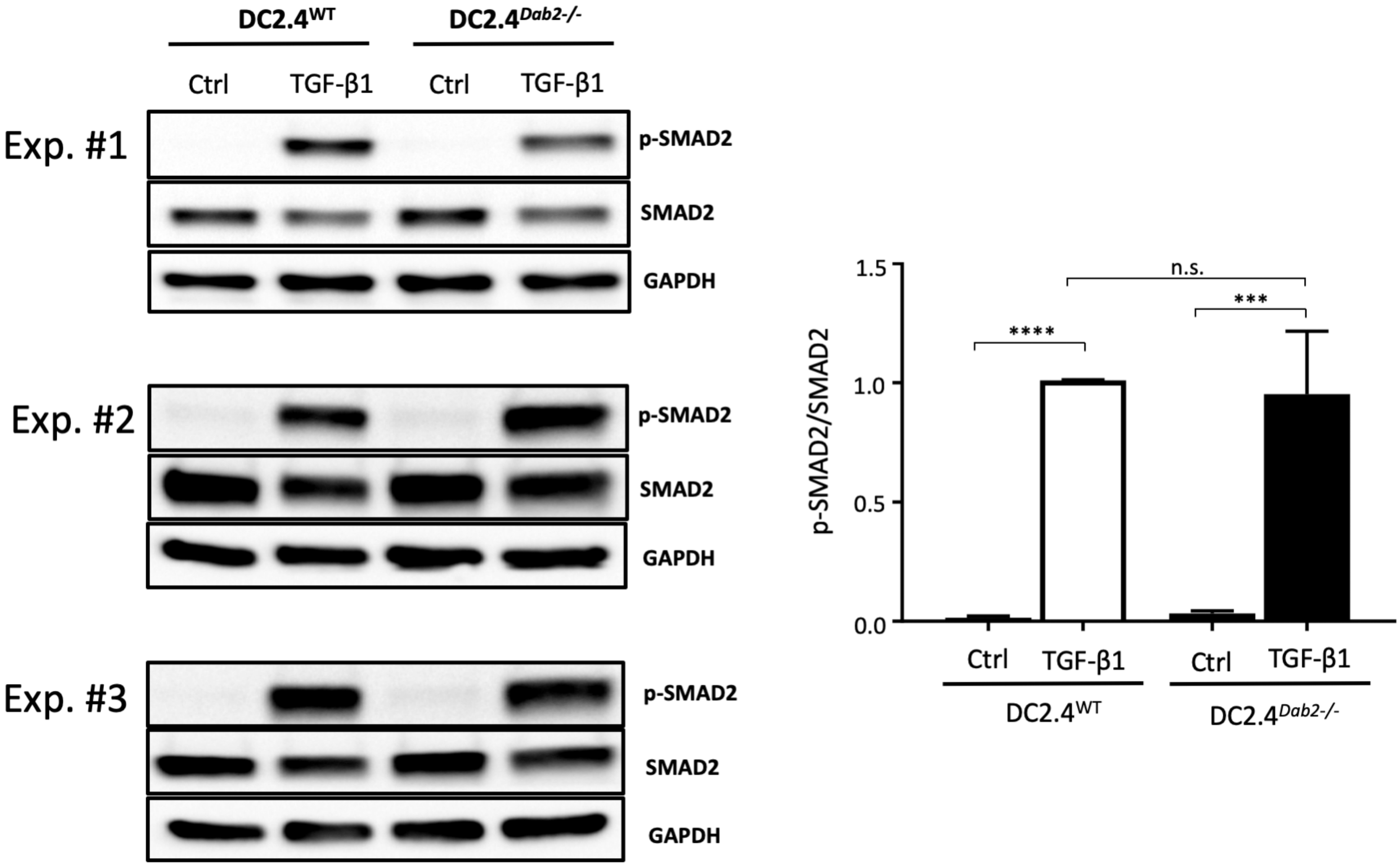

Fig. S8. Lack of *Dab2* expression does not impact the canonical TGF-β signaling in dendritic cells. DC2.4^{WT} and DC2.4$^{Dab2-/-}$ cells were incubated with 10 ng/mL TGF-β1 for 1 hour and phosphorylated SMAD2 (p-SMAD2; pSer^{465}/Ser^{467}) or total SMAD2 were evaluated by western blotting. Due to inter-experimental variation (passage number) in SMAD2 expression, representative Western blotting results from three independent experiments are shown. Summary data is shown in the bar graph (n = 3; ANOVA followed by Tukey post-hoc test, *** p<0.001, **** p<0.0001; n.s. – not significant).
