# Supplementary material for: Rapid Downregulation of DAB2 by Toll-Like Receptor Activation Contributes to a Pro-Inflammatory Switch in Activated Dendritic Cells
Source: Front Immunol. 2019 Feb 27;10:304. doi: 10.3389/fimmu.2019.00304 (PMC6400992; doi:10.3389/fimmu.2019.00304)
Supplement: Supplementary file 2 [file Data_Sheet_2.PDF]

Table S1. Primers used for qRT-PCR

**Target**	**Assay Id**	**Source**
MMP-8	Mm00439509_m1	ThermoFisher Scientific
MyD88	Mm00440338_m1	ThermoFisher Scientific
TRAF-6	Mm00493836_m1	ThermoFisher Scientific
Ticam-1/TRIF	Mm00844508_s1	ThermoFisher Scientific
IRF-3	Mm00516784_m1	ThermoFisher Scientific
IL-10	Mm00439614_m1	ThermoFisher Scientific
IL-17A	Mm00439618_m1	ThermoFisher Scientific
IL-6	Mm00446190_m1	ThermoFisher Scientific
TNF	Mm00443258_m1	ThermoFisher Scientific
IL-12a	Mm00434165_m1	ThermoFisher Scientific
IL-4	Mm00445259_m1	ThermoFisher Scientific
IL-23a	Mm01160011_g1	ThermoFisher Scientific
IL-1β	Mm00434228_m1	ThermoFisher Scientific
TBP	Mm00446971_m1	ThermoFisher Scientific
IFN-β1	Mm00439552_s1	ThermoFisher Scientific
TLR4	Mm00445274_m1	ThermoFisher Scientific
CXCL1	Mm00433859_m1	ThermoFisher Scientific
IFN-ɣ	Mm00801778_m1	ThermoFisher Scientific
Dab2	Mm01307290_m1	ThermoFisher Scientific

Table S2. Antibodies used for flow cytometry

**Antibody**	**Clone**	**Fluorochrome**	**Source**
CD11c	N418	PE	eBioscience
CD103 (Integrin alpha E)	2E7	PerCP-eFluor® 710	eBioscience
CD11b	M1/70	efluor 450	eBioscience
F480	BM8	BV510	Biolegend
CD80	B7.1	APC	eBioscience
MHC Class II (I-A/I-E)	M5/114.15.2	efluor 450	eBioscience
CD40	3/23	PE	BDBiosciences
CD184 (CXCR4)	L276F12	APC	Biolegend
CD199 (CCR9)	eBioCW-1.2	PE	eBioscience
CD197 (CCR7)	4B12	PE	eBioscience
CD45R (B220)	RA3-6B2	FITC	eBioscience

Table S3. Antibodies used for western blot

**Antibody**	**Clone**	**Host**	**Source**
Dab2	D7O9T	Rabbit	Cell Signaling
Phospho-IκBα (Ser32)	14D4	Rabbit	Cell Signaling
LC3A/B	D3U4C	Rabbit	Cell Signaling
p-ULK1 (S555)	D1H4	Rabbit	Cell Signaling
Total ULK-1	D8H5	Rabbit	Cell Signaling
SQSTM1/p62	-	Rabbit	Cell Signaling
Caspase-3	8G10	Rabbit	Cell Signaling
Cleaved Caspase-8 (Asp387)	D5B2	Rabbit	Cell Signaling
β-actin	AC-74	Mouse	Sigma Aldrich
GAPDH	1D4	Rabbit	ThermoFisher Scientific
